# Advances in Targeting HPV Infection as Potential Alternative Prophylactic Means

**DOI:** 10.3390/ijms22042201

**Published:** 2021-02-23

**Authors:** Sinead Carse, Martina Bergant, Georgia Schäfer

**Affiliations:** 1 International Centre for Genetic Engineering and Biotechnology (ICGEB) Cape Town, Observatory 7925, South Africa; crssin001@myuct.ac.za; 2 Institute of Infectious Disease and Molecular Medicine (IDM), Faculty of Health Sciences, University of Cape Town, Observatory 7925, South Africa; 3 Division of Medical Biochemistry and Structural Biology, Department of Integrative Biomedical Sciences, Faculty of Health Sciences, University of Cape Town, Observatory 7925, South Africa; 4 Laboratory for Environmental and Life Sciences, University of Nova Gorica, Vipavska 13, 5000 Nova Gorica, Slovenia; martina.bergant@ung.si

**Keywords:** human papillomavirus (HPV), cervical cancer, low- and middle- income countries (LMIC), prophylactic vaccines

## Abstract

Infection by oncogenic human papillomavirus (HPV) is the primary cause of cervical cancer and other anogenital cancers. The majority of cervical cancer cases occur in low- and middle- income countries (LMIC). Concurrent infection with Human Immunodeficiency Virus (HIV) further increases the risk of HPV infection and exacerbates disease onset and progression. Highly effective prophylactic vaccines do exist to combat HPV infection with the most common oncogenic types, but the accessibility to these in LMIC is severely limited due to cost, difficulties in accessing the target population, cultural issues, and maintenance of a cold chain. Alternative preventive measures against HPV infection that are more accessible and affordable are therefore also needed to control cervical cancer risk. There are several efforts in identifying such alternative prophylactics which target key molecules involved in early HPV infection events. This review summarizes the current knowledge of the initial steps in HPV infection, from host cell-surface engagement to cellular trafficking of the viral genome before arrival in the nucleus. The key molecules that can be potentially targeted are highlighted, and a discussion on their applicability as alternative preventive means against HPV infection, with a focus on LMIC, is presented.

## 1. Introduction

Papillomaviruses (PVs) are small, non-enveloped, double stranded DNA viruses belonging to the highly diverse family of Papillomaviridae [1,2]. Over 200 types of human papillomaviruses (HPVs) alone have been characterized [3] and are classified as either high-risk or low-risk, depending on their ability to cause malignancy [1]. HPVs infect the skin and mucosal epithelial cells, causing hyper-proliferative lesions. Mucosal HPV types are highly prevalent and are transmitted through sexual activity. This leads to a significant disease burden on a global scale as persistent infection with high-risk (oncogenic) HPVs are associated with 5% of all human cancers. Of those, the most common HPV-associated malignancy is cervical cancer with virtually all cases linked to an underlying oncogenic HPV infection [4,5]. Over 20 oncogenic HPV types have been identified and characterized, out of which HPV16 and 18 are the most common types in the world and contribute to 70% of all documented cases [4,6]. Low-risk HPV types, such as HPV6 and 11, do not cause cancer. However, infection with these HPV types can result in the manifestation of benign lesions and warts of the anogenital areas [7]. Moreover, perinatally acquired HPV6 and 11 can cause recurrent respiratory papillomatosis in infants and young children [7].

Cervical cancer is more prevalent in low-and middle-income countries (LMIC), accounting for more than 80% of the global disease burden [8]. World-wide, cervical cancer ranks fourth for both incidence and mortality in women [9]. However, it is the second most common cancer in South Africa [9,10] as well as the leading cause of female cancer-associated deaths in Sub-Saharan Africa [11]. The higher incidence of HPV and cervical cancer in LMIC has not least been attributed to the Human Immunodeficiency Virus (HIV)/acquired immunodeficiency syndrome (AIDS) epidemic in these regions [12], and in fact, cervical cancer is considered an AIDS-defining malignancy [13].

As infection with oncogenic HPVs is almost always the cause of cervical cancer, it is potentially an entirely preventable condition. However, the current therapies aim to remove lesions and abnormal cells rather than targeting the HPV infection as the causative agent [14]. To date, no direct anti-HPV treatment is available. However, three highly efficacious prophylactic vaccines exist against the most common high-risk HPV types [15,16,17]. All three vaccines provide protection against HPV16 and 18, with Gardasil-9 (Merck) additionally protecting against new infection with the oncogenic types 31, 33, 45, 52, and 58 [16]. However, the vaccines do not provide treatment for individuals who are already infected with HPV. Since all three HPV vaccines are composed of virus-like particles (VLPs) derived from the spontaneous assembly of individual type-specific L1 capsid proteins [18], they provide little to no cross-protection against other HPV types that are not included in the vaccines as the immune response is highly type specific. This means that women remain exposed to high-risk HPVs that are not covered by these vaccines and develop low- and high-grade cervical intraepithelial lesions [19]. A concerning and unexplained phenomenon is also that women vaccinated with first-generation (bivalent and quadrivalent) HPV vaccines are at higher risk of contracting non-HPV 16/18 high-risk HPV types than unvaccinated women [20].

Although the South African Department of Health began a vaccination program and rolled out Cervarix™ to school girls (who are approximately 9 years of age) in 2014 [10], vaccinations are unlikely to alleviate the burden of HPV-related diseases in the near future. The need to receive multiple vaccination shots as well as the limited access in health care workers reaching the designated schools are believed to be the main reasons why many young girls are thought to have missed the opportunity to be immunized. Furthermore, the need for a cold chain (controlled storage of the vaccine at low temperatures) has been difficult to maintain in LMICs with substandard infrastructure and low funding, which makes administration of the vaccines to schoolgirls in rural areas even more challenging [21]. Since the current vaccination programs are limited to girls in a specific year of schooling, a large number of young girls will not have had the opportunity to receive the vaccine through this program, and the high costs of the vaccines limit their affordability to a high proportion of the population [22]. This is further exacerbated by the stigma and lack of knowledge surrounding cervical cancer, HPV infection, and HPV vaccination, as well as reproductive health in general, as adolescent health platforms often do not exist [21,22].

Given that the disease burden resulting from persistent HPV infections is likely to continue, alternative therapies for the treatment and prevention of HPV infections are required, particularly ones that are more easily accessible to people in LMIC. Topical antiviral microbicides that can block the full spectrum of genital HPV infections (as well as other common sexually transmitted infections (STIs)) could either complement the current vaccines, be offered to those without access to these vaccines, or even be given to people who are already infected with HPV. In 2001, Christensen et al. noted the lack of research into compounds with microbicidal activity against papillomaviruses [23]. The only reagents that had been studied and shown to inhibit HPV infection were monoclonal antibodies (MAbs) with type-specific neutralizing activity, and broad-spectrum antiviral agents such as alkyl sulfates and monocaprin [24,25,26,27]. Over the last two decades, research has delved into discovering and characterizing a broad spectrum of molecules with anti-HPV activity. Many approaches are targeting the early steps of HPV infection (cell-surface binding, intracellular trafficking, etc.) as a promising strategy in preventing and treating HPV infections.

This review briefly summarizes the current knowledge on the early stages of HPV infection, from cell surface binding to intracellular trafficking, and discusses some of the research in the discovery and development of anti-HPV molecules, with the aim to provide an overview of alternative prophylactic means that could be potentially developed in HPV/cervical cancer prevention strategies. 

## 2. Early Stages of HPV Infection

In order to develop molecules that target the early steps of HPV infection, the mechanisms used by HPV for internalization and successful infection must be understood. This review will provide a brief summary of HPV entry. For more comprehensive reviews of papillomavirus cell surface binding and entry, refer to Raff et al. [28] and Day et al. [29]. For in depth reviews on papillomavirus endocytosis, refer to Siddiqa et al. [30] and Mikuličić et al. [31].

HPV displays a rather specific target cell tropism and primarily infects the basal keratinocytes of squamous epithelium, such as is found in the mucosal epithelial lining of the cervix [32,33]. It is well established that epithelial wounding (or damage) is a pre-requisite for efficient HPV infection in an in vivo setting [34,35,36]. It is likely that abrasions in the outermost protective layers help the virions gain access to the mitotically-competent basal epithelial cells in the inner layers of the epithelium, as these are the cells that are able to establish episomal viral genome replication [34,37]. 

HPVs encode two capsid proteins that make up the viral structure and are essential for host cell engagement and the early steps in infection. The major capsid protein (L1) spontaneously assembles into a 72-pentamer icosahedral structure [38,39]. The minor capsid protein (L2) is not essential for capsid formation but plays an important role in successful infection [40]. A maximum occupancy of 72 L2 molecules can be incorporated into the capsid of an HPV particle [40]. 

### 2.1. HSPG Binding at The Cell Surface

Both the major L1 capsid protein and the mainly hidden minor L2 capsid protein have been shown to be involved in the initial binding and entry steps into the target cells of the basal epithelium [39,41]. However, the exact mechanisms and surface receptors used by HPV for successful entry and subsequent establishment of infection are still debated.

**Figure 1:**
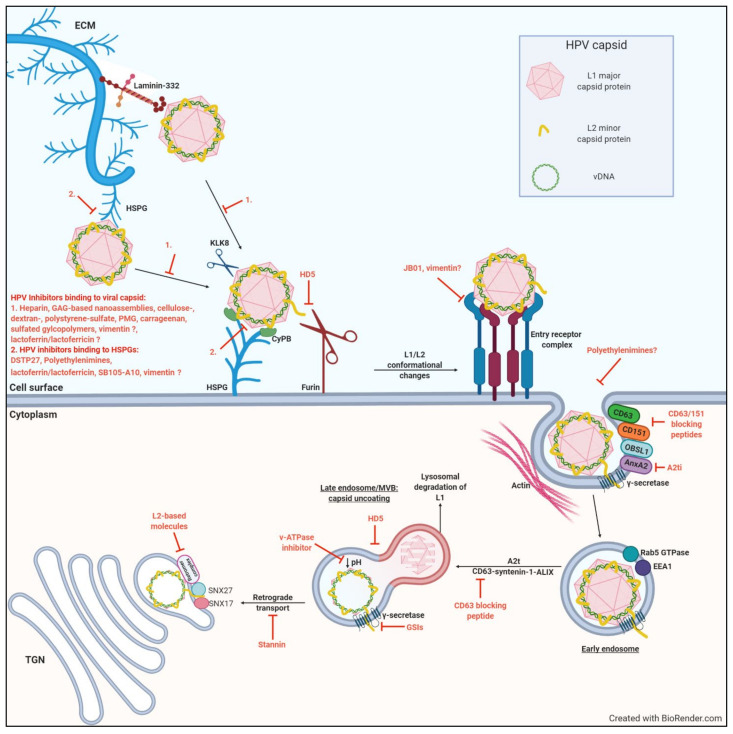
Schematic illustration of the early stages of human papillomavirus (HPV) infection, indicating the sites of inhibition of various anti-HPV molecules. Briefly: binding to heparan sulfate proteoglycans (HSPGs) results in conformational changes of the viral capsid, which facilitates interactions between the capsid and cyclophilin B (CyPB) and cleavage by kallikrein-8 (KLK8). This results in the exposure of the L2 N-terminus, containing a conserved site which is cleaved by furin. This is followed by dissociation of the capsid from HSPGs and exposure of a binding site in L1, potentially needed for recognition by an unknown entry receptor complex. The viral capsid undergoes ligand induced endocytosis (which is clathrin-, caveolin-, cholesterol- and dynamin-independent) and depends on the reorganization of the actin cytoskeleton. Membrane protrusion of the L2 capsid protein is chaperoned by γ-secretase, which allows L2 to interact with host cell factors in the cytosol during transport. HPVs localize with early antigen 1 (EEA10) compartments in a Rab5 GTPase-dependent manner. The early endosome matures into the late endosome when it fuses with lysosomes. Lysosomal degradation is driven by the formation of multivesicular bodies (MVBs). Most of the contents are degraded by lysosomal degradation, including the majority of the L1 capsid protein. Endosomal escape and subsequent post-endocytic trafficking of high-risk HPVs is regulated by CD63-syntenin-1-ALIX, which traffics HPV to these multivesicular endosomes. Here, the capsids disassemble in a pH-dependent manner, and egress from the late endosome. The L2/vDNA cargo enters the TGN, where the protruding L2 protein interacts with SNX17, SNX27 and retromer complex.

It is widely accepted that the initial cell surface molecules that HPVs interact with are heparan sulphate proteoglycans (HSPGs) [34,42,43]. HSPGs are glycoproteins that are found on the cell surface and in the extracellular matrix (Figure 1), where they interact with a wide range of ligands and enhance formation of their receptor-signaling complexes [44,45]. In mammals, syndecans and glypicans constitute the predominant cell surface HSPGs [46,47]. HSPGs are negatively charged molecules comprising of a core protein linked to glycosaminoglycan (GAG) chains of unbranched sulfated polysaccharides known as heparan sulfates (HS). HS are structurally related to heparin, but heparin is more sulfated and has a higher content of iduronic acid [48]. Many infectious pathogens and viruses interact with HSPGs on the cell surface, or on the basement membrane, for attachment and subsequent cellular entry, making it a promising target for viral inhibitors [49,50,51,52,53,54,55,56]. Specifically, the L1 capsid protein of HPV binds to the GAG chains of HSPGs [34].

Apart from HSPG binding, HPV has been proposed to bind to laminin 332 (a protein secreted into the extracellular matrix by migrating keratinocytes) in a transient manner, which results in uptake by proliferating keratinocytes expressing α6 integrin [57,58].

### 2.2. Furin Cleavage and Subsequent Transfer to An Unknown Receptor/Receptor Complex

Binding to HSPGs induces conformational changes in the capsid and facilitates proteolytic cleavage of L1 by the secreted serine-protease kallikrein-8 (KLK8) [34,59]. This cleavage allows for interactions between the capsid and cyclophilin B, which results in further conformational changes that exposes the L2 N-terminus (Figure 1). The exposed N-terminus contains a conserved consensus cleavage site for the host extracellular proprotein convertase furin [60]. This interaction has been shown to be essential for successful infection of HPV, as furin cleavage results in the exposure of a binding site on L1, postulated to be recognized by an unknown receptor, or receptor complex [34,59,61]. The described changes in virion conformation further facilitate the reduction in the binding affinity to HSPGs, thereby facilitating the engagement with the unknown receptor(s), or receptor complex (Figure 1) [62]. The importance of furin for successful infection was demonstrated by using furin precleaved HPV-PsVs together with HSPG deficient cell lines: only furin precleaved PsVs were able to infect HSPG-deficient cells, while untreated PsVs could not [63]. These findings highlight the role of initial HSPG attachment to facilitate the critical step of L2 cleavage by furin and association with the putative second receptor/receptor complex for entry. Furin cleavage has also been implicated in successful endosomal escape prior to transport of the L2/viral DNA complex to the nucleus, emphasizing the necessity of furin cleavage for successful HPV infection [29,61]. 

### 2.3. Intracellular Trafficking and Transport of The L2/Vdna Complex to The Nucleus

After the binding of HPV to the unknown receptor/receptor complex, the capsids undergo internalization via endocytosis [30]. The mode of HPV internalization has been contradictorily discussed. No generalized mechanism for HPV endocytosis has been reported, as it seems to depend on host cell and virus type [30]. More recent report describes a novel endocytic pathway exploited by HPV16 during host cell entry. This ligand-induced pathway is clathrin-, caveolin-, cholesterol-, and dynamin-independent and depends on the reorganization of the actin cytoskeleton, thereby sharing many requirements with macropinocytosis but differing in the mode of vesicle formation [64,65,66]. Other studies revealed that tetraspanin CD151 and possibly other tetraspanins [67], the cytoskeletal adaptor obscurin-like 1 (OBSL1) [65,68], and the phospholipid-binding protein annexin A2 [31,69] are also involved in HPV16 endocytosis (Figure 1). It has been shown that HPV16, 18, and 31 share similar requirements for entry [70], suggesting that all HPV types of the alpha genus use this endocytosis pathway [31]. 

The endocytosed HPVs are sorted into early endosomes, and HPV16 and 31 have been reported to localize with early endosome antigen 1 compartments in a Rab5 GTPase-dependent manner [71,72]. The early endosome matures into the late endosome, when it fuses with lysosomes [30]. Most of the contents are degraded by lysosomal degradation, including a majority of the L1 capsid protein [64,66]. The L2/viral DNA (L2/vDNA) complex, however, escapes the endosome for further trafficking towards the nucleus [30]. The driving force for lysosomal degradation of HPV-containing late endosomes is the formation of multivesicular bodies (MVBs) which contain intralumenal vesicles (ILVs) [30]. The post-endocytosis trafficking of high-risk HPVs is regulated by CD63-syntenin-1-ALIX, which has been shown to traffic HPV16, 18 and 31 to multivesicular endosomes, where the capsids disassemble, leading to exposure and transport of the L2/vDNA complex to the trans golgi network (TGN) [73]. Annexin A2 heterotetramer (A2t) has also been implicated in virus progression to MVBs, where the depletion of A2t reduces capsid uncoating and an apparent increase in lysosomal degradation of L1 [74]. 

Late endosome sorting and the subsequent disassembly of the HPV capsid is pH-dependent [30,75]. Endosomal acidification assists in separating the L2/vDNA from the L1 during capsid uncoating. However, recent studies have shown that a small amount of intact L1 remains associated with the L2/vDNA complex and accompanies the complex to the TGN and nucleus (Figure 1) [76]. 

As previously mentioned, furin-cleaved L2 is crucial for successful HPV infection as it is instrumental in endosomal escape. Specifically, the C-terminal cell penetration peptide (CPP) of L2 that protrudes through the endosomal membrane initiates membrane insertion of the viral particle, while its N-terminal putative transmembrane (TM) domain then reaches the membrane bilayer [77,78,79]. γ-Secretase has been proposed as a chaperone that interacts with the L2 TM domain to promote its membrane insertion, where the N-termiinal region upstream of the TM remains in the lumen throughout the cellular entry process after insertion [80]. After the L2/vDNA cargo egresses from the late endosome, it enters the TGN, where the vDNA remains in a vesicular compartment and a small portion of the L2 protein is accessible in the cytosol during transport for interactions with the host cell factors, such as SNX17, SNX27, and retromer complex [30]. Retromer complex, which is involved in the retrograde transport of different cargos, is critical for retrograde trafficking of the HPV16 L2/vDNA cargo to the TGN (Figure 1) [81]. VAP dependent ER-endosome contact might be involved in cleavage of virion containing vesicles and facilitating the trafficking to the TGN, as extensive VAP dependent endosomal tabulation has been reported to be induced by HPV-16 [82].

Cell cycle progression and subsequent nuclear envelope break down is another crucial step in HPV infection [83,84]. Several groups demonstrated that the main restriction on nuclear entry of the L2-vDNA complex and the initiation of viral gene expression is the completion of mitosis [85,86]. The L2/vDNA complex therefore remains in the TGN until it is transported to the nucleus [30]. After the onset of mitosis, the Golgi and TGN undergo fragmentation and vesiculation, and L2/vDNA-containing vesicles then egress from the TGN and associate with microtubules [30,87]. The vesicles migrate along the microtubules towards the condensed chromosomes, where it has been suggested that the L2 protein interacts with the chromosomes to ensure the L2/vDNA-containing vesicles remain at the mitotic spindle [88]. Finally, L2/vDNA complex localize to punctate nuclear foci called promyelocytic leukemia (PML) bodies, or ND10 (nuclear domain 10), in the interphase of the infected cells [84,89], where the viral DNA transcription is believed to start.

Potential target sites preventing the entry process are indicated by red block arrows with the inhibitory molecules described in detail in the text.

## 3. Molecular Targets of Early HPV Infection

The inability to propagate HPV in vitro has hampered research into the early stages of HPV infection. However, by expressing the L1 protein using a number of eukaryotic expression systems, it is possible for L1 to self-assemble into virus-like particles [38,90]. Co-expression of L1 and L2 along with a reporter gene to measure infection [91,92,93] has, for example, led to the development of HPV pseudovirions (HPV-PsVs) which has been widely used to study HPV entry mechanisms as well as to test potential inhibitory molecules of HPV.

### 3.1. Targets of Heparan Sulfate Binding 

The main objective in the development of microbicides against HPV (or any viral infection) is to block the interaction between the virion proteins and the cell surface receptors that are used by the virus to gain entry into the cells. As discussed previously, initial HSPG binding is an important step to successful HPV internalization (Figure 1), as its inhibition has shown to decrease HPV infection in vitro and in vivo [42,43,62]. Since several different viruses use HS moieties as the initial receptor/coreceptor to bind to the cell surface (see Section 2.1), it is considered a viable drug target, particularly with regards to producing a microbicide that has broad spectrum protection against a number of HPVs (as well as other sexually transmitted viruses).

#### 3.1.1. Heparin and Heparin-Based Molecules

The interaction between HPV and HSPGs occurs between the basic amino acid residues of viral proteins and the negatively charged sulfated/carboxyl groups of the GAG chains [48]. Because of this, heparin and other GAGs can competitively interfere with virus attachment to cells [42,94,95]. Indeed, HPV11 VLPs were shown to interact with heparin and with cell-surface GAGs in vitro, with high molecular weight heparin inhibiting infection with a half maximal inhibition concentration (IC_50_) of 14.9 µM [95]. A commercial low molecular weight heparin was also tested and was shown to have an IC_50_ of 762 µM [95]. Giroglou et al. confirmed that heparin also readily bound to HPV16 and 33-PsVs, and the complete suppression of infection was observed at a heparin concentration of 2 µM (0.05 mg/mL) in vitro [42]. Other glycosaminoglycans, such as dermatan sulfate and chondroitin sulfate, had no significant effect on HPV33-PsV infection [42]. Day et al. has later shown that treatment of HPV16-PsVs with 10 µg/mL of soluble heparin prevented cell surface association with HaCaT cells in vitro, resulting in an accumulation of the virus on the ECM [96]. This again suggests that heparin acts as a competitive inhibitor that binds to viral particles, blocking binding of the virions to surface HSPGs. 

However, HPV’s sensitivity to heparin may differ depending on the HPV production system used, as organotypic raft-culture derived HPV16 was found to attach to the cell surface in the presence of heparin, while HPV18, 31 and 45 were unable to bind HaCaT cells [97]. Although HPV 31 and 45 were unable to interact with the cell surface in the presence of heparin, they were resistant to inhibition [97]. This has previously also been seen for HPV31b infection of HaCaT cells [98] and suggests that under certain experimental conditions, HPV16, 31 and 45 may attach to cells using a non-HS receptor, and that different HPV types may utilize different molecules for initial attachment to the basement membrane [97]. The discrepancy in the effect of heparin between organotypic derived or pseudovirion production HPV particles may partly be explained by the presence of cellular factors and proteases, which are necessary for infection (see Section 2.2.), being present in the differentiating tissue culture, while being mostly absent from the pseudovirus system. 

Even more surprising is a finding published by Cerqueira et al. in 2013, where preincubation of HPV16-PsVs with increasing concentrations of heparin resulted in partial restoration rather than more efficient inhibition of infection in vitro [99]. Moreover, interaction of virions with heparin allowed HPV16-PsV infection in the absence of cell surface HSPGs. From these findings, it seems as if heparin acted in lieu of HSPGs to allow eventual secondary receptor binding and internalization [99]. Therefore, there may not be an absolute requirement for a specific HSPG receptor for HPV16, but rather specific glycan moieties [99]. 

These studies warrant the research of modified heparin molecules or other heparin-like molecules that have the same mechanism of action: binding to multiple HPV types and preventing HSPG binding. Moreover, the effects of heparin in an in vivo model should be explored for the different HPV types, as cell culture systems do not accurately mimic the effects in a complex physiological and/or clinical context.

Research into drug delivery systems based on GAGs targeting viral infections led to the development of spontaneously forming GAG-based nanoassemblies, in the hopes to incorporate them into locally administered formulations to target viruses, to load antiviral drugs, and to control their release over time [48]. The preparations of these spontaneous nanoassemblies are formed in aqueous medium and do not require surfactants, pH modification, or any heating and purification steps. In this process, nanoassemblies are obtained by mixing a hydrophobically-modified polysaccharide and an α-cyclodextrin (α-CD). The preparation of heparin-based nanoassemblies was achieved by the self-association of O-palmitoyl-heparin (OPH) and α-CD. This resulted in the formation of well-structured hexagonal-shaped nanoassemblies that could specifically bind and “trap” viruses and avoid their attachment to GAGs on the cell surface [48]. The effect of the chemical modification of OPH on antiviral activity was evaluated against HPV16-PsVs in vitro. The most potent nanoassembly inhibited HPV16-PsVs with an IC_50_ of 1.25 µg/mL [48]. 

#### 3.1.2. Naturally Derived Sulfated Polysaccharides

Several high-molecular-weight sulfated/sulfonated polysaccharides or polymers have been shown to have microbicidal activity against a number of sexually transmitted infections, such as HIV and HSV-1 and 2 [36,100,101,102,103]. Therefore, their microbicidal activity against papillomaviruses has been assessed. Indeed, dextran sulfate (DS) and polystyrene sulfate (PSS) showed strong inhibition of bovine papillomavirus (BPV), HPV11 and HPV40 in vitro with IC_50_ values ranging from 10 to 100 µg/mL [23]. Cellulose sulfate (CS), however, required greater concentrations to achieve virus inactivation (1000 µg/mL) [23]. The authors proposed that DS and PSS achieved virus inactivation by binding to either the virus or cell surface, thereby blocking virus attachment [23]. PSS, however, inhibited HPV11 infection even after the virus had attached to the cell surface. This result suggested that blocking attachment to the cell surface may not be the exclusive mechanisms of inhibition of these compounds [23]. 

Alginate, a soluble acidic polysaccharide found in brown seaweeds, composed of a central backbone of poly-d-mannuronic acid (PM), poly-l-guluronic acid (PG), and alternate residues of d-mannuronic acid and l-guluronic acid (PMG), was shown to significantly inhibit HPV16- and HPV18-PsVs infection of HEK-293FT, HeLa and HaCaT cells, with IC_50_ values ranging between 0.7 and 3.3 µg/mL [104]. Further studies with HPV45-PsVs indicated that PMGs may block the very early steps of the viral life cycle by directly associating with the L1 protein [104]. Using a murine skin infection model, the researchers showed a significant reduction in HPV45-PsVs infection when particles were pretreated with PMGs (10 mg) or when PMGs were administered during infection. Inhibition by alginate has also been documented for HIV, HSV and Hepatitis B virus (HBV), providing evidence for the broad spectrum antiviral capabilities of brown seaweed-derived polysaccharides [105,106,107].

An important sulfated polysaccharide that has already entered clinical trials in the prevention of HPV infection is carrageenan. It was first identified as a highly potent inhibitor of HPV6, 16, 18, 31 and 45-PsVs in 2006, using high-throughput screening [108]. The study showed that carrageenan treatment in the low ng/mL range resulted in 50% inhibition [108]. Extracted from red algae, carrageenan has also been shown to inhibit HSV and HIV infections in vitro [109]. The primary mode by which carrageenan blocks HPV infection is by directly binding to the viral capsid, thereby blocking its interactions with cell surface HSPG attachment factors [109]. Carrageenan was also shown to have an HSPG-independent effect, either by blocking interactions with cellular proteins involved in the internalization process or by preventing conformational changes to the capsid that are necessary for infectious internalization [36]. However, depending on the experimental system used, conflicting results are reported in the literature (see also Section 3.1.1.); while carrageenan was shown to be effective in inhibiting HPV18 and HPV31 native viral infections, HPV16 and HPV45 could not be blocked by carrageenan, even at high carrageenan concentrations (100 mg/mL) [97]. These two HPV types are suggested to use GAG-independent mechanisms to infect the host cell. Therefore, different carrageenan compounds/derivates need to be considered for broad-spectrum antiviral activities against HPV. 

Several groups explored the potential of carrageenan-based lubricants/microbicides in inhibiting HPV infection [109]. A study by Roberts et al. proved carrageenan’s in vivo application, where two commercial carrageenan-containing lubricants (Divine 9 and BIOglide) decreased HPV16-PsV infection in a mouse model [36]. A separate study tested Divine 9 and the Population Council’s PC-515 gel against HPV16, 18, and 45-PsV infection in a mouse model. Inhibition of infection was achieved for all HPV types tested, particularly with PC-515, proving carrageenan’s broad-spectrum anti-HPV activity in vivo [110]. Novetsky et al. performed a phase I clinical study to evaluate the inhibition of HPV16-PsV infection following the use of ‘Divine 9,’ a commercial 2% carrageenan-containing lubricant [111]. The study involved the collection and testing of cervicovaginal lavages in vitro in two groups of women without prior HPV vaccination or cervical intraepithelial neoplasia. The first group was instructed to insert one dose of Divine 9 within 12 h before vaginal intercourse; the second group was instructed to do the same but to additionally administer a second dose of gel as soon as possible after intercourse. Cervicovaginal lavage (CVL) samples were combined with HPV16-PsVs to assess for inhibition of viral infection in HEK-293TT cells. As a result, 93% of CVL samples showed HPV16-PsV inhibition with a median of 97.5%, decreasing slightly over time [111]. Higher carrageenan concentrations were associated with higher HPV16-PsV inhibition [111]. In 2011, Marais et al. reported a negative association of HPV infection with a vaginal 3% carrageenan-based microbicide (Carraguard^®^), based on a phase III trial, comparing compliant Carraguard^®^ users and compliant placebo users [112]. Carrageenan was found to be associated with a 38% protective effect, but only among the most compliant participants. This randomized, double-blind, placebo-controlled trial conducted in South Africa was originally designed to assess the efficacy of a carrageenan-based gel in reducing the risk of HIV infection in women, where the study did not show Carraguard^®^’s efficacy in the prevention of vaginal transmission of HIV [112,113]. 

Two more recent clinical trials have been published in 2019, assessing the effectiveness of carrageenan-based gels on HPV infection [114,115]. Magnan et al. conducted a randomized, double-blind, placebo-controlled trial to assess the efficacy of a carrageenan-based lubricant gel in reducing genital HPV incidence and prevalence among sexually active women [114]. Two groups, one receiving the carrageenan lubricant and the other a placebo lubricant, were instructed to self-apply inside the vagina and on the genital area every second day for the first month and before and after each intercourse. The carrageenan-based gel was associated with a 36% protective effect compared to the placebo, thereby reducing the risk of both low and high oncogenic risk genital HPV infections in women [114]. Perino et al. performed an observational study on the use of Carvir gel, a new carrageenan/proprionibacterium extract-based vaginal microbicide [115]. This study recruited 40 fertile females with genital HPV infections with (group A), or without (group B) associated HPV-related low-risk genital lesions. Although the main outcome of the report was to evaluate the safety and satisfaction of the use of the new gel, the authors reported early findings on the rate of HPV genital infection clearance at the end of follow-up in the intervention population, comparing these data with spontaneous clearance in a control group. At the final follow-up visit, 60% of all patients had become HPV negative (57.7% of group A; 64.3% of group B); among these, 13 cases were high-risk HPV infections. Patients treated with the carrageenan-based gel showed an HPV clearance five-times higher (*p* = 0.005) than the control group [115]. The study, however, did not determine the effectiveness of the carrageenan-based gel in the clearance of a specific papillomavirus genotype [115].

#### 3.1.3. Synthetic Sulfated Polysaccharides

It is argued that the use naturally derived sulfated polysaccharides for safe clinical application faces a number of challenges, not the least of which is the possibility of spontaneous/alternative modifications in these natural compounds leading to heterogeneity and impurities [108,116]. Most recently, the potential of highly sulfated glycomimetics as inhibitors of HPV binding and infection has been assessed [116]. Glycomimetics are synthetic glycan analogues which can even exceed the activities of the natural polysaccharides from which they are derived [117]. These synthetic analogues have enhanced stability, bioavailability, and half-life, making them ideal molecules to explore in their capacity as antivirals.

Soria-Martinez et al. designed and synthesized both glycopolymers (long-chain) and glycooligomers (short-chain) with a high degree of sulfation and a polymeric display of saccharides (features shared with both heparin and carrageenan) [116]. Two different glycopolymers were synthesized, one incorporating galactose side chains (PG1 and PG2) and the other mannose side chains (PM). PG and PM had degrees of polymerization (dp) of 40–44 and 86 respectively, and subsequently underwent sulfation. The pre-incubation of HPV16-PsVs with either PG1 or PM abolished infection at 0.01 mg/mL in vitro, providing evidence that a dp of 40 is enough to achieve maximal blocking efficacy. Their unsulfated counterparts could not inhibit infection. PM and PG1/2 exhibited IC_50_ values of 7 × 10^−5^ and 2 × 10^−4^ mg/mL respectively, which is as efficient as carrageenan and up to 100-fold more efficient than heparin. The glycooligomers synthesized were much shorter than the glycopolymers and GAGs. Overall, they were far less potent than the glycopolymers in inhibiting HPV16-PsV infection, with an average IC_50_ of 0.1 mg/mL in vitro. Microscopic analysis showed that glycopolymers blocked binding of HPV16-PsVs to cell surface HS. Unexpectedly, glycooligomers allowed binding but not infection, suggesting a novel manner in which these sulfated polymers can interfere with viral infection [116]. A mouse vaginal challenge model was used to test the effectiveness of the oligopolymers in vivo. PG2 was able to completely block infection in all mice, proving the in vivo antiviral effects of sulfated glycopolymers [116]. 

#### 3.1.4. Dispirotripiperazine

An alternative strategy to prevent virus binding to cellular HSPGs is the use of compounds that interact with HS chains, thus competitively inhibiting virus attachment (Figure 1). Dispirotripiperazine (DSTP), a class of low-molecular-weight antiherpetic compounds, was shown to bind to heparin, preventing binding of viral particles to HS moieties on the cell surface [118]. The N,N’-bisheteryl derivative of dispirotripiperazine, DSTP27 has been reported by Selinka et al. to block HPV16 and 18-PsV infection over an extended period of time, both pre- and post-binding of virions to the cell surface [62]. Firstly, it was reported that DSTP27 binds to cell surface HS preventing the initial binding of virions to the cell surface. Secondly, DSTP27 was shown to prevent transfer from the primary attachment receptor to a non-HSPG uptake receptor. DSTP27 efficiently blocked HPV16 and 18-PsV infection of HEK 293TT cells with IC_50_ values of approximately 0.8 and 0.4 µg/mL respectively, and significantly reduced HPV infection for more than 30 h post infection [62]. It was further proposed that in addition to the primary interaction of HPV with HSPGs, the secondary interaction partners of HPV virions must also include HSPG molecules, since both heparinase and DSTP27 prevented transfer from the primary attachment receptor to a non-HSPG uptake receptor [62]. 

#### 3.1.5. Polyethylenimines

Polyethylenimines (PEIs) are cationic polymers with known antimicrobial activity [119]. Given their polycationic nature, PEIs can condense with DNA, resulting in PEI-DNA complexes which mediate HSPG-dependent gene transfer into mammalian cells in vitro and in vivo [120]. Since PEIs interact with surface-expressed HSPGs, their potential use as competitive HPV inhibitors was assessed [119]. Indeed, the infection efficiency of HPV16-PsVs was significantly reduced by PEI in a concentration-dependent manner in vitro, with 52nM PEI inhibiting HPV16-PsV infection by more than 99%. No cytotoxic effects were exhibited at this concentration (CC_50_: 856.7 nM). Similar results were seen for HPV-PsV types 18 and 31 across multiple cell lines. It was further confirmed that PEIs affect the primary attachment of viral particles as PEI treatment almost completely blocked viral attachment to the cell surface [119]. Importantly, PEI-mediated inhibition 24 h post infection was maintained when administered more than 24 h before and up to 4 h after addition of pseudovirions. This result suggests that PEI not only inhibits the binding process of HPV16 but might also interfere with post binding events (Figure 1) [119].

#### 3.1.6. Lactoferrin/Lactoferricin

Lactoferrin (LF), a monomeric glycoprotein found in secretions like saliva, breastmilk, and semen, has important roles in host defense, and has been shown to inhibit a number of fungi, bacteria, and viruses [121], such as HSV, HCMV, hepatitis C virus (HCV), poliovirus, HIV, and HPV [122,123,124,125,126]. In 2004, Drobni et al. reported LF to act early in the HPV16 VLP uptake process, potentially on the receptor binding step, as the antiviral effect of LF gradually disappeared in cells treated with HPV16 VLPs for increasing lengths of time before LF treatment [127]. The study focused on both human derived LF (hLF) and bovine derived LF (bLF) and reported that bLF had the more potent inhibitory effect on HPV16 VLP uptake, with 50% inhibition at doses of 35 µg/mL [127]. A subsequent study showed hLF and bLF had a strong inhibitory effect on HPV5- and HPV16-PsVs infection (IC_50_ values of 0.32 ± 0.18 µM and 0.25 ± 0.24 µM respectively) [128]. A number of human and bovine lactoferricins (peptides released by lactoferrin pepsin digestion) have also been shown to have antiviral activity [129]. A set of defined derivatives were analyzed, where derivative bLfcin 17–3 showed the most potent inhibition of HPV5 and HPV16-PsV infection in vitro, with IC_50_ values in the low µM range for all cell lines analyzed [128].

Glycosaminoglycan chains on the cell surface have been reported to interact with LF [130], suggesting that LF binding to heparan sulfate blocks HPV receptor binding (Figure 1). However, LF could also inhibit HPV uptake by directly binding to the virions, as has been reported for poliovirus [131].

#### 3.1.7. Dendrimers

Dendrimers are large, artificial, highly branched macromolecules synthesized from a polyfunctional core. They can incorporate peptide bonds such as repeat units of polyamino acids or polyethers that completely react with the functional groups of the core, in turn leaving terminal functional groups that can react again [132]. Dendrimer molecules have been synthesized to contain functional groups in the surface layer that can form complexes with cell or viral receptors, disrupting normal virus-cell interactions, including the initial binding of virus to the cell [132]. 

In an attempt to identify antiviral molecular antagonists of HSPGs, Donalisio et al. screened a library of linear, dimeric, and dendrimeric peptides containing clusters of basic amino acids that could bind to the negatively charged sulfate and carboxyl groups of HS, thus inhibiting initial phases of HPV infection (Figure 1) [133]. SB105-A10 was one such peptide which bound to the surfaces of two different epithelial cell lines, with EC_50_ values between 53 to 85 nM. Importantly, no cytotoxicity was observed [133]. In vitro analysis identified SB105-A10 as a potent inhibitor of HPV-PsV types 16, 18 and 6, with IC_50_s ranging between 0.59 and 0.88 µM. In addition, SB105-A10 retained its inhibitory activity even when it was added to cell cultures 2 h after PsV infection, suggesting a post-attachment inactivation mechanism [133]. The finding that SB105-A10 is active even against multiple HPV types suggests a broad-spectrum capability of this molecule against papillomaviruses. Besides its anti-HPV activity, SB105-A10 also proved active against HIV-1 infection in vitro [133]. 

### 3.2. Targets of HPV Cellular Internalization 

As mentioned previously, the exact mechanisms and host cellular receptors used by HPV for successful internalization remain elusive. However, a few host cellular factors that are involved in successful endocytosis of viral particles have been documented. Some have been researched as potential targets of HPV infection [31,69].

#### 3.2.1. Vimentin

Vimentin is a type II intermediate filament found in mammalian cells [134,135,136]. Vimentin plays a role in a variety of cellular functions ranging from integrating into the entire cytoskeleton, responding to mechanical stress, cellular adhesion, to signaling [137,138]. Although best described as a cytosolic protein involved in cellular processes such as cell adhesion and cell migration, vimentin has been identified on the surface as well as in the extracellular spaces of cells of various origins [139,140,141,142]. Many reports have been published describing vimentin’s role in viral infection, with the majority showing vimentin’s role in facilitating infectious internalization, such as Japanese encephalitis virus, Human Cytomegalovirus and Enterovirus 71 [143,144,145,146]. 

In an attempt to identify key host molecules that interact with HPV16-PsVs, cell-surface vimentin was identified as a novel HPV-binding molecule [41]. Surprisingly, vimentin overexpression in multiple cell lines resulted in decreased levels of infection, while vimentin knockout by siRNA led to an increase in infection. Moreover, pre-incubation of HPV16-PsVs with soluble recombinant human vimentin resulted in a decrease in viral internalization by more than 50% in vitro. This suggested a vimentin-mediated blocking of virus attachment and cellular entry, although the exact molecules involved in the vimentin-mediated inhibition of HPV entry was not elucidated (Figure 1). However, infection as measured by luciferase activity 48h later was not affected, possibly due to dissociation of the vimentin-virus complexes [41]. The latter result warrants the exploration into stabilizing the association of vimentin with viral particles in order for vimentin to exhibit prolonged inhibition of HPV infection.

#### 3.2.2. Anhydride-modified Protein (JB01)

Several groups have explored the ability of 3-hydroxyphthalic anhydride-modified bovine beta-lactoglobulin, termed JB01, to potently inhibit HIV and HSV-1 and -2 [147,148,149]. In 2013, Lu et al. reported that this anhydride-modified protein can also inhibit the entry of HPV in vitro [150]. JB01 showed potent inhibition of HPV-PsV types 6, 16 and 18, with IC_50_ values of 0.33, 0.04, 0.065 mM, respectively [150]. Also, HPV58-PsV entry in vitro was found to be inhibited by JB01 with an IC_50_ of 0.28 μg/mL [151]. Mechanistically, the negatively charged regions on JB01 were shown to bind to the positively charged region of the HPV L1 protein, and this interaction was proposed to competitively block the binding of HPV to the receptor on the basement membrane in the vaginal mucosa (Figure 1) [151].

Importantly, the cytotoxicity of JB01 was determined to be insignificant in both human cervical cells and vaginal epithelial cells, and could not be detected in the blood samples of rhesus macaques, suggesting its safe use in a clinical setting [150]. In a randomized clinical trial published in 2016, topical application of a vaginal gel containing JB01 showed significant efficacy and safety in the treatment of high-risk HPV infections [152]. In this study, women aged 25–65 years old infected by high-risk HPV, such as HPV types 16, 18, 31, 33, 35, 39, 45, 51, 52, 56, 58, 59 and 68 were recruited. After 3 months of treatment, about 60.5% of HPV-positive women in the treatment group became HPV-negative compared with 13.5% of women in the non-treatment group [152]. Importantly, JB01 was found to be highly stable for up to 12 weeks, even at the human body temperature (37 °C) [150]. 

#### 3.2.3. Annexin A2 

As mentioned previously, annexin A2 facilitates infectious entry of HPV16 into epithelial cells [31]. HPV16 particles interact with annexin A2 in association with S100A10 as a heterotetramer at the cell surface in a calcium-dependent manner [69]. The annexin A2/S100A10 heterotetramer (A2t) was shown to facilitate HPV16 entry through a direct protein–protein interaction between the S100A10 subunit of A2t and the HPV16 L2 [153]. Woodham et al. saw this interaction as an opportunity to explore the effects of an annexin A2 inhibitor, A2ti, on HPV16 infection in vitro [154]. Indeed, A2ti reduced HPV16-PsV infection in HeLa and HaCaT cells in a dose dependent manner, with a 100% inhibition of infection at 100 µM. Additionally, A2ti significantly decreased HPV16-PsV entry, with a 65% reduction at 100 µM. This result indicated that the inhibition of HPV16-PsV infection with A2ti is due in part to a block in viral entry (Figure 1) [154].

#### 3.2.4. Tetraspanin Blocking Peptides (CD63 and CD151)

A number of tetraspanins have been implicated in the successful entry of HPV particles into target cells [31,67]. In 2018, Fast et al. determined which specific tetraspanin domains were involved in HPV infection, particularly for CD63 and CD151 [67]. Of particular interest was the C-terminal region, as this region facilitates complex formation between CD63 and its direct interaction partner syntenin-1 [155], a requirement for post-endocytic trafficking of HPV [73]. HPV infection also involves the C-terminus of CD151, as deletion of this region causes CD151 to lose its activity with HPV16 [156]. Therefore, cytopermeable peptides comprising the C-terminal region sequence of tetraspanin were assessed with regard to their potential inhibitory functions. In vitro assays showed a dose dependent decrease in HPV16-PsV infection in HeLa and HaCaT cells treated with the peptides (IC_50_ values ranging from 3.64 and 9.16 µM) [67]. A reduction in HCMV infection was also seen, and it was proposed that these peptides may inhibit the entry of other viruses. Using immunofluorescence assays, it was found that the CD63 and CD151 C-terminal peptides decreased the disassembly of HPV16 capsids, which consequently inhibited infection, and that the inhibitory effect of C-terminal CD63 and CD151 peptides occurs prior to HPV capsid disassembly conforming to the role of CD63 and CD151 during HPV entry (Figure 1) [67].

### 3.3. Targets of Intracellular Trafficking

Once internalized, endosomes containing HPV particles undergo maturation. The formation of multivesicular bodies (MVBs) result in the sorting and subsequent disassembly of the HPV capsid. This is followed by endosomal escape of the L2/vDNA complex, where it is translocated to the host cell nucleus [30,157]. Factors regulating endosomal escape and post-endocytosis trafficking of high-risk HPVs are also potential targets of HPV infection and can be exploited to inhibit infection. 

#### 3.3.1. v-ATPase Inhibitors

Endosomal/lysosomal acidification is necessary for successful infection by a number of viruses, including HPV [64,158]. Vacuolar ATPases (v-ATPases) which acidify the late endosome by pumping protons across the endosomal membrane [159], were shown to be required for successful HPV infection due to their role in the viral uncoating step (Figure 1) [158]. Consequently, a number of different v-ATPase inhibitors were tested for antiviral activity against HPV infections. Of those, Saliphenylhalamide (SaliPhe), a derivative of the naturally occurring compound salicylihalamide, has proven to be a potent inhibitor of mammalian v-ATPases [160]. SaliPhe was found to be an effective inhibitor against HPV 6, 11, 16, 18 and 31-PsV infection in vitro, with 50% inhibition in the nM range [158]. This was considered indicative of broad-spectrum anti-HPV activity of this group of compounds.

#### 3.3.2. Human α-Defensin 5

Using a high-throughput PsV-based screen, human α-defensin 5 (HD5) was identified as a highly potent human innate antimicrobial peptide with inhibitory activity against genital HPV types 6, 16, 18, and 31, with similar IC_50_ values of approximately 0.6 µg/mL [161]. It was further suggested that HD5 blocks virion escape from endocytic vesicles but not virion binding or internalization [161]. Indeed, Wiens and Smith showed that HD5 interacts directly with HPV16-PsVs and inhibits the furin-mediated cleavage of L2 at the cell surface during infection at a step downstream of the cyclophilin B-mediated unfolding of L2 [162], thereby affecting endosomal escape. Interestingly, furin precleaved HPV16-PsVs were still blocked by HD5, suggesting an additional mode of inhibition. HD5 was found to inhibit HPV16-PsV infection by adversely affecting the dissociation of the major capsid protein L1 and the viral genome which is critical for productive infection (Figure 1) [30]. HD5 treatment therefore dramatically changes the trafficking of the viral genome and capsid proteins downstream of the early endosome, redirecting them away from the TGN and to the lysosome. Based on these two studies, it is likely that the way in which HD5 interacts with the capsid to block L2 cleavage also precludes the separation of L1 and L2 either by maintaining overall capsid integrity or by stabilizing a subviral complex of L1, L2,and genome [162]. 

##### 3.3.3. γ-Secretase Inhibitors

The requirement of γ-secretase for successful infection is a unique feature of HPVs [163,164]. γ-secretase is a transmembrane protease, which is involved in cleavage of transmembrane domains of numerous membrane proteins. Knockdown of any of the four subunits leads to a potent block of HPV infection [163]. In 2010, Huang et al. showed that γ-secretase inhibitors (GSIs) inhibited the infectivity of HPV16-PsVs in human keratinocytes at non-cytotoxic doses with IC_50_ in the picomolar to nanomolar range [165]. Similar results were seen for HPV11 and 31-PsVs. A strong inhibition of HPV infection was observed also in the mouse model using topically-applied GSIs, suggesting that GSIs could represent effective microbicides against anogenital HPVs [165]. Sensitivity to GSI was later confirmed in diverse alpha and beta HPV types in vitro, with sensitivity greater than that of furin inhibition [166]. Although the exact mechanism is unknown, it appears that inhibition of γ-secretase activity results in a failure of L2/vDNA to reach the TGN (Figure 1), which is consistent with the observation that HPV16 is only sensitive to γ-secretase inhibition during the first 6–8 h of infection [164]. 

#### 3.3.4. Stannin

Stannin, an 88-residue transmembrane protein, has been proposed to affect HPV16-PsV infection in a similar manner to HD5. Using an overexpression genetic screen for genes that affect HPV16-PsV infection, the *SNN* gene was identified that resulted in about a 50% decrease in HPV5, 16 and 18-PsV infection in vitro [157]. Moreover, SNN deficient mutant cell lines resulted in a 50% increase in HPV16-PsV infection to that of their wildtype counterparts [157]. Further experiments showed that stannin does not affect virus uptake or virus uncoating, but instead blocks virus entry into the TGN by routing the cargo to lysosomal compartments for degradation. Stannin overexpression reduced L2 and VPS35 co-localization, suggesting that it abrogates L2-retromer binding, a critical step for L2/vDNA to enter the TGN (Figure 1) [30,157].

#### 3.3.5. L2-Based Molecules

The L2 capsid protein has been implicated in participating in a series of downstream subcellular trafficking events to ensure the nuclear delivery of the HPV genome. Therefore, inhibiting intracellular virus trafficking by targeting L2 functions has been considered. Yan et al. confirmed the importance of a highly conserved 36-amino-acid peptide sequence of the L2 N terminus (L2N) for HPV infection [77]. This region contains the furin cleavage sequence (RTKR) (17), an RG1/JWW-1 cross-type neutralization epitope with the conserved cysteine residues for intramolecular disulfide bonds (17, 38), and a putative TM domain adjacent to the glycine-rich GXXXG motif [79]. Indeed, ectopic expression of this L2N terminal region on the cell surface greatly reduced HPV 16-PsV infection in various cell lines [77]. Lipidation of the L2N peptide (creating L2N lipopeptides) successfully mimicked the transmembrane domain function and blocked TGN trafficking of HPV, leading to rapid virion degradation. As furin cleavage was necessary for the lipopeptides to inhibit infection, it was speculated that the L2 N-terminal region interacts with a yet unknown host membrane protein necessary for the successful endosomal escape of HPV particles.

Further research into L2 targeting peptides revealed that a peptide (P16/16) containing the C-terminal region of HPV16 L2, spanning the L2 cell penetrating peptide (CPP) and the adjacent retromer binding site (RBS)) not only inhibited HPV16-PsV infection, but HPV5 and 18-PsVs as well [167]. P16/16 was shown to enter cells from the culture medium and bind retromer, thereby sequestering it from incoming HPV. This is proposed to prevent endosome exit and trafficking of the incoming virus to the TGN, thereby aborting infection (Figure 1) Moreover, P16/16 showed a decrease in infection in a mouse model with marginal statistical significance (*p* = 0.088), proving that the peptide was not inactivated in the female reproductive tract and can access basal keratinocytes in live tissue [167].

## 4. Conclusions

The current statistics on HPV and cervical cancer prevalence alone highlight the need for alternative therapies for the prevention and treatment of HPV infections. The existing HPV vaccines, although highly efficacious, do not provide protection against all the high-risk HPV types, much less the low-risk HPV types. Furthermore, these vaccines are purely prophylactic and are not easily accessible for women in LMIC. For these reasons, alternative therapies that have broad spectrum protection against HPV types, as well as other sexually transmitted infections are worth exploring. Many LMIC countries, especially those in Sub Saharan Africa are highly burdened with the HIV/AIDS pandemic, where co-infection with HPV leads to accelerated and often more severe HPV-induced dysplasia and cancer due to progressive immune suppression. Therefore, a microbicide capable of protecting against HPV and HIV would be the most beneficial in an LMIC setting. Cost-effective means of production and affordability by women in LMIC are also important aspects to consider. 

Targeting the early stages of viral entry is particularly promising and has been extensively researched (Figure 1, Table 1). The knowledge of the viral particle components and the host cell entry factors and their specific interactions can enable the design of efficient antiviral strategies. One such target is inhibiting the initial attachment of viral particles to the cell surface by either binding to the viral particle or by interacting with the attachment receptor itself. The anti-HPV molecules that follow this mechanism of inhibition have also been shown to inhibit other viral infections (Table 1). While it seems that most HPV and other viruses use HSPGs during initial attachment to their target cells, the exact mechanisms and conditions for successful attachment of the various virus (sub)types remain speculative. Furthermore, the specific uptake receptors or receptor complexes used by HPV subsequent to attachment are not known. This should not deter further research into the molecules that target HPV in this manner, but rather promote further investigation. 

We have already seen success in one such molecule, carrageenan, in reducing infection in a clinical setting (Table 1). Carrageenan is already commercially produced and has the potential to provide women a cost-effective protective alternative against HPV infection. Carraguard^®^, a carrageenan-based microbicide, has shown to be safe to use. Unfortunately, it has also been shown to be ineffective in protecting against HIV. 

Many of the molecules targeting attachment detailed in this review have only been tested with PsVs in vitro. Important to note are also the different approaches taken by these studies, and how discrepancies in experimental design can yield different results. For instance, the use of PsVs as opposed to native viral particles can yield conflicting results. PsVs, although it has shed much light on the early steps of HPV infection, may not provide the accurate picture needed to design highly effective inhibitors of HPV uptake in vivo. Different HPVs may use different strategies to attach to and infect their host, as is evident by some of the molecules described in this review having differential inhibition amongst different HPV types. These are some of the few important factors to consider when developing new agents to block HPV infections. 

Furthermore, targeting downstream intracellular trafficking also looks promising, where many of the molecules described in this review not only demonstrated inhibition of HPV infections, but other sexually transmitted infections as well (Table 1). There is much merit in researching molecules that target common stages of the viral life cycle, particularly by targeting the virus–host interaction that are shared by most HPV types as well as other infections such as HSV and HIV. However, efficient intracellular delivery is more difficult to obtain compared to substances blocking viral attachment at the cell surface.

The development of microbicides that contain one or a combination of the antiviral molecules described here may provide many women with easily accessible protection against sexually transmitted infections. Health care workers could be trained in the use and importance of microbicides and can advocate microbicides for their ease of use. The development of microbicides that can be sold over the counter (much like condoms) could have a substantial impact against common STIs, especially if it were used by a significant number of women. However, certain challenges might be anticipated in the development and distribution of antiviral microbicides, not least of which is the production cost. To maintain its affordability to women in an LMIC setting, the antiviral molecule production system must be low cost and highly scalable so that it can be introduced and manufactured in LMICs. These microbicides will need to be stabilized for storage for prolonged periods at room temperature as cold storage is often inaccessible. Furthermore, it is important to establish a sustainable supply of these microbicides before creating a demand for them. Funding and successful clinical trials may also prove a challenge, which creates further issues in establishing the safety and efficacy of these microbicides. If antiviral microbicides reach the stage for safe use, the distribution of the microbicide as well as all the necessary information must be appropriate for an LMIC setting. Women in LMIC may not have easy access to distribution points for microbicides (and other methods of protection), nor the necessary information on its use, safety and efficacy and appropriate hygiene standards that should be implemented along with its use. Widespread access through a range of distribution points (clinics, pharmacies, grocery stores) should be considered, together with sexual and reproductive health education.

## Figures and Tables

**Table 1:** Summary of potential inhibitory molecules that target the early steps of HPV infection.

Potential Inhibitory Molecule	Proposed Mechanism of Inhibition	Affected HPV Type	Experimental System Used, Stage of Clinical Trial if Applicable	Inhibitor of Other Sexually Transmitted Infections	Caveats/Challenges	Ref
**Heparin**	Competitive inhibitor, binds to viral capsids and prevents capsid binding to HSPGs	11 VLP	In vitro: HaCaT, CHO-K1, pgsA-745 cells	HIV-1 [168,169,170,171] HSV-1/2 [56] HCMV [172]	Use of PsV vs. organotypic derived HPV led conflicting results; HPV16-PsV interaction with heparin shown to aid infection in the absence of cell-surface HSPGs	[95]
		16, 33 PsV	In vitro: COS-7, HeLa, DG75 cells			[42]
		16 PsV	In vitro: HaCaT cells			[96]
		18 NV (* 16, 31, 45 NV)	In vitro: HaCaT, CHO par, pgsA-745, primary human keratinocytes derived from newborn foreskin			[97]
**GAG-based nanoassemblies: O-palmitoyl-heparin (OPH)**		16 PsV	In vitro: 293TT cells	HSV-1/2 [48]		[48]
**Cellulose, dextran, polystyrene sulfate**		11, 40 NV	In vitro: A431 cells	HIV [100,173] HSV-1/2 [100,102,173] *Chlamydia* [101,102] *Neisseria gonorrhoeae* [101,102]		[23]
**Alginate (PMG)**		16, 18, 45 PsV	In vitro: 293FT, HeLa, HaCaT cells In vivo: cutaneous PsV infection in mice	HIV [107] HSV [105] HBV [106]		[104]
**Carrageenan**		6, 16, 18, 31, 45 PsV	In vitro: HeLa, HaCaT, 293TT, C127 cells	HIV [174] HSV [175]		[108]
		18, 31 NV (* 16, 45 NV)	In vitro: COS-7, HeLa, DG75 cells			[97]
		16 PsV	Human samples used in vitro: 293TT cells			[111]
		16 PsV	In vivo: mouse cervicovaginal challenge model			[36]
		16, 18, 45 PsV	In vivo: mouse cervicovaginal challenge model			[110]
		16, 18, 31, 33, 35, 39, 45, 51, 52, 56, 58, 59, 68 detected	Phase III clinical trial			[112]
		low oncogenic risk: 6, 11, 40, 42, 44, 54 high and intermediate oncogenic risk: 16, 18, 26, 31, 33, 34, 35, 39, 45, 51, 52, 53, 56, 58, 59, 66, 67, 68, 69, 70, 73, 82 commensal types: 61, 62, 71, 72, 81, 83, 84, 89	Phase 2B clinical trial		No indication of heterogeneity of effects when stratifying by validated vaccination status. No indication of a dose-response relationship with the estimated cumulative compliance.	[114]
		6, 11, 16, 18, 31, 33, 39, 40, 42, 45, 51, 52, 53, 55, 56, 58, 59, 61, 62, 66, 73, 81, 84	Prospective observational clinical study		Single-centered study without randomization of patients. Effectiveness on clearance of a specific papillomavirus genotype no evaluated. Short study observation period.	[115]
**Sulfated glycopolymers**		16 PsV	In vitro HeLa cells: In vivo: mouse vaginal challenge model	HSV-1 [116]		[116]
**Vimentin**	Inhibitor of virus attachment	16 PsV	In vitro: HeLa, HaCaT, CHO-K1, pgsD-677, NIKS cells			[41]
**Dispirotripiperazine (DSTP27)**	Binds to HSPGs, blocking capsid binding to the cell surface	16, 18 PsV	In vitro: 293TT, CHO-K1, pgsA-745, HaCaT cells	HSV-1/2 [118] HIV-1 [118] HCMV [118]		[62]
**Polyethylenimines**		16, 18, 31 PsV	In vitro: HeLa, Cos7, HaCaT, 293TT, pgsA-745, CHO-K1 cells	HCMV [119]		[119]
**Lactoferrin/lactoferricin**		16 VLP	In vitro: HaCaT cells	HSV-1/2 [122] HCMV [176] HIV-1 [177] HBV [178]		[127]
		5, 16 PsV	In vitro: HaCaT, C33A cells			[128]
**Dendrimers (SB105-A10)**		6, 16, 18 PsV	In vitro: SiHa, HeLa, C33A, HL3T1, 293TT, CHO-K1 cells	HIV-1 [179] HCMV [180] HSV-1/2 [181,182]		[133]
**Anhydride modified protein (JB01)**	Inhibits viral entry, binds to L1	6, 16, 18 PsV	In vitro: 293FT cells	HIV [183] HSV-1/2 [147,148]		[150]
		16, 58 PsV	In vitro: HeLa, HaCaT cells			[151]
		16, 18, 31, 33, 35, 39, 45, 51, 52, 56, 58, 59, 68	Phase I/IIa clinical trial			[152]
**Annexin 2 heterotetramer inhibitor (A2ti)**	Inhibits viral entry, binds to A2ti	16 PsV	In vitro: HeLa, HaCaT			[154]
**Tetraspanin blocking peptides (CD63 and CD151)**	Inhibits viral entry, blocks tetraspanin functions	16 PsV	In vitro: HeLa, HaCaT cells	HCMV [67]		[67]
**v-ATPase inhibitor (SaliPhe)**	Inhibition of Lysosomal acidification, viral uncoating targeted	6, 11, 16, 18 PsV	In vitro: HeLa HaCaT, 293TT, NHEK cells			[158]
**Human α-defensin 5 (HD5)**	Inhibits furin mediated cleavage; Disrupts capsid dissociation	16 PsV	In vitro: HeLa, HaCaT, 293TT, C127 fibroblast cells	HSV-1/2 [184]		[161]
		16 PsV	In vitro: HeLa, HaCaT cells			[185]
**γ-secretase inhibitors (GSIs)**	Prevents L2/vDNA from reaching the TGN	11, 16, 31 PsV	In vitro: HaCaT, C127, HeLa cells In vivo: mouse cervicovaginal challenge model			[165]
**Stannin**	Blocks virus entry into TGN	16 PsV	In vitro: HeLa, HaCaT cells			[157]
**L2-based molecules**	Block TGN trafficking	16 PsV	In vitro: HeLa, CHO-K1, 293T, Huh7, U251, pgsA-745 cells			[77]
		16 PsV	In vitro: HeLa, HaCaT cells			[78]

*indicates HPV types that are not affected. Legend: VLP: virus-like particle; **PsV:** pseudovirus **NV:** native virus; **HPV:** papillomavirus; **HIV:** Human Immunodeficiency Virus; **HSV:** herpes simplex virus; **HCMV:** human cytomegalovirus; **HBV:** hepatitis B virus; **TGN:** trans golgi network.

## Data Availability

The data that support the findings of this article are openly available at PubMed (https://pubmed.ncbi.nlm.nih.gov/ (accessed on 3 February 2021)).

## References

[1] De Villiers E.M., Fauquet C., Broker T.R., Bernard H.U., Zur Hausen H. (2004). Classification of papillomaviruses. Virology.

[2] Zheng Z.M., Baker C.C. (2006). Papillomavirus genome structure, expression, and post-transcriptional regulation. Front. Biosci..

[3] International Human Papillomavirus (HPV) Reference Center Human Reference Clones. https://www.hpvcenter.se/human_reference_clones/.

[4] Bosch F.X., Manos M.M., Muñoz N., Sherman M., Jansen A.M., Peto J., Schiffman M.H., Moreno V., Kurman R., Shan K.V. (1995). Prevalence of Human Papillomavirus in Cervical Cancer: A Worldwide Perspective. J. Natl. Cancer Inst..

[5] Zur Hausen H. (2002). Papillomaviruses and cancer: From basic studies to clinical application. Nat. Rev. Cancer.

[6] Munoz N., Bosch F.X., Castellsague X., Diaz M., De Sanjose S., Hammouda D., Shah K.V., Meijer C.J.L.M. (2004). Against Which Papillomavirus Types Shall We Vaccinate and Screen? The International Perspective. Int. J. Cancer.

[7] Lacey C.J.N., Lowndes C.M., Shah K.V. (2006). Chapter 4: Burden and management of non-cancerous HPV-related conditions: HPV-6 / 11 disease. Vaccine.

[8] International Agency for Research on Cancer (2012). WHO Cervical Cancer: Estimated Incidence, Mortality And prevalence Worldwide in 2012.

[9] Bray F., Ferlay J., Soerjomataram I. (2018). Global Cancer Statistics 2018: GLOBOCAN Estimates of Incidence and Mortality Worldwide for 36 Cancers in 185 Countries. CA Cancer J. Clin..

[10] (2017). World HPV Information Center Human Papillomavirus and Related Diseases Report-South Africa. ICO HPV Inf. Cent. Rep..

[11] De Vuyst H., Alemany L., Lacey C., Chibwesha C.J., Sahasrabuddhe V., Banura C., Denny L., Parham G.P. (2013). The Burden of Human Papillomavirus Infections and Related Diseases in Sub-Saharan Africa. Vaccine.

[12] Williamson A.-L. (2015). The Interaction between Human Immunodeficiency Virus and Human Papillomaviruses in Heterosexuals in Africa. J. Clin. Med..

[13] Cobucci R.N.O., Lima P.H., de Souza P.C., Costa V.V., da Conceição de Mesquita Cornetta M., Fernandes J.V., Gonçalves A.K. (2015). Assessing the impact of HAART on the incidence of defining and non-defining AIDS cancers among patients with HIV/AIDS: A systematic review. J. Infect. Public Health.

[14] Stanley M.A. (2012). Genital human papillomavirus infections: Current and prospective therapies. J. Gen. Virol..

[15] Siddiqui M.A.A., Perry C.M. (2006). Vaccine (Gardasil ®). Drugs.

[16] Zhai L., Tumban E. (2016). Gardasil-9: A global survey of projected efficacy. Antiviral Res..

[17] Monie A., Hung C.F., Roden R., Wu T.C. (2008). Cervarix^TM^: A vaccine for the prevention of HPV 16, 18-associated cervical cancer. Biol. Targets Ther..

[18] Stanley M., Lowy D.R., Frazer I. (2006). Chapter 12: Prophylactic HPV vaccines: Underlying mechanisms. Vaccine.

[19] Heley S., Brotherton J. (2009). Abnormal Pap tests after the HPV vaccine. Aust. Fam. Physician.

[20] Brown D.R., Kjaer S.K., Sigurdsson K., Iversen O.E., Mauricio H.A., Wheeler C.M., Perez G., Koutsky L.A., Tay E.H., Garcia P. (2009). The impact of quadrivalent human papillomavirus (HPV; Types 6, 11, 16, and 18) L1 virus-like particle vaccine on infection and disease due to oncogenic nonvaccine HPV types in generally HPV-naive women aged 16-26 years. J. Infect. Dis..

[21] Denny L. (2015). Control of Cancer of the Cervix in Low- and Middle-Income Countries. Ann. Surg. Oncol..

[22] Tathiah N., Chb M.B., Man D.H.I.V., Epi M.S., Phm M. (2015). Human papillomavirus (HPV) vaccination of adolescents in the South African private health sector: Lessons from the HPV demonstration project in KwaZulu-Natal. South Afr. Med. J..

[23] Christensen N.D., Reed C.A., Culp T.D., Hermonat P.L., Howett M.K., Anderson R.A., Zaneveld L.J.D., Hemother A.N.A.G.C. (2001). Papillomavirus Microbicidal Activities of High-Molecular-Weight Cellulose Sulfate, Dextran Sulfate, and Polystyrene Sulfonate. Antimicrob. Agents Chemother..

[24] Christensen N.D., Kreider J.W., Cladel N.M., Patrick S.D., Welsh P.A. (1990). Monoclonal Antibody-Mediated Neutralization of Infectious Human Papillomavirus Type 11. J. Virol..

[25] Christensen N.D., Cladel N.M., Reed C.A. (1995). Postattachment Neutralization of Papillomaviruses by Monoclonal and Polyclonal Antibodies. Virology.

[26] Howett M.K., Neely E.B., Christensen N.D., Wigdahl B., Krebs F.C., Malamud D., Patrick S.D., Pickel M.D., Welsh P.A., Reed C.A. (1999). A Broad-Spectrum Microbicide with Virucidal Activity against Sexually Transmitted Viruses. Antimicrob. Agents Chemother..

[27] Howett M.K., Wigdahl B., Malamud D., Christensen N.D., Wyrick P.B., Krebs F.C., Catalone B.J. Alkyl sulfates: A new family of broad spectrum microbicides. Proceedings of the XIII International AIDS Conference. Monduzzi Editore, International Proceedings Division (Bologna, Italy).

[28] Raff A.B., Woodham A.W., Raff L.M., Skeate J.G., Yan L., Da Silva D.M., Schelhaas M., Kast W.M. (2013). The Evolving Field of Human Papillomavirus Receptor Research: A Review of Binding and Entry. J. Virol..

[29] Day P.M., Schiller J.T. (2009). The role of furin in papillomavirus infection. Future Microbiol..

[30] Siddiqa A., Broniarczyk J., Banks L. (2018). Papillomaviruses and Endocytic Trafficking. Int. J. Mol. Sci..

[31] Mikuličić S., Florin L. (2019). The endocytic trafficking pathway of oncogenic papillomaviruses. Papillomavirus Res..

[32] Stanley M.A. (2012). Epithelial Cell Responses to Infection with Human Papillomavirus. Clin. Microbiol. Rev..

[33] Chow L.T., Broker T.R., Steinberg B.M. (2010). The natural history of human papillomavirus infections of the mucosal epithelia. Authors J. Compil..

[34] Ozbun M.A. (2019). Extracellular events impacting human papillomavirus infections: Epithelial wounding to cell signaling involved in virus entry. Papillomavirus Res..

[35] Shope B.R.E., Hurst B.E.W. (1933). Infectious Papillomatosis of Rabbits. J. Exp. Med..

[36] Roberts J.N., Buck C.B., Thompson C.D., Kines R., Bernardo M., Choyke P.L., Lowy D.R., Schiller J.T. (2007). Genital transmission of HPV in a mouse model is potentiated by nonoxynol-9 and inhibited by carrageenan. Nat. Med..

[37] Stanley M. (2010). HPV-immune response to infection and vaccination. Infect. Agent. Cancer.

[38] Kirnbauer R., Booy F., Cheng N., Lowy D.R., Schiller J.T. (1992). Papillomavirus L1 major capsid protein self-assembles into virus-like particles that are highly immunogenic. Proc. Natl. Acad. Sci. USA.

[39] Buck C.B., Thompson C.D., Pang Y.-Y.S., Lowy D.R., Schiller J.T. (2005). Maturation of Papillomavirus Capsids. J. Virol..

[40] Buck C.B., Cheng N., Thompson C.D., Lowy D.R., Steven A.C., Schiller J.T., Trus B.L. (2008). Arrangement of L2 within the Papillomavirus Capsid. J. Virol..

[41] Schäfer G., Graham L.M., Lang D., Blumenthal M.J., Bergant M., Katz A.A. (2017). Vimentin modulates infectious internalisation of HPV16 pseudovirions. J. Virol..

[42] Giroglou T., Florin L., Schafer F., Streeck R.E., Sapp M. (2001). Human Papillomavirus Infection Requires Cell Surface Heparan Sulfate. Am. Soc. Microbiol..

[43] Johnson K.M., Kines R.C., Roberts J.N., Lowy D.R., Schiller J.T., Day P.M. (2009). Role of Heparan Sulfate in Attachment to and Infection of the Murine Female Genital Tract by Human Papillomavirus. J. Virol..

[44] Sarrazin S., Lamanna W.C., Esko J.D. (2011). Heparan sulfate proteoglycans. Cold Spring Harb. Perspect. Biol..

[45] Bernfield M., Götte M., Park P.W., Reizes O., Fitzgerald M.L., Lincecum J., Zako M. (1999). Functions of Cell Surface Heparan Sulfate Proteoglycans. Annu. Rev. Biochem..

[46] Bernfield M., Kokenyesi R., Kato M., Hinkes M., Spring J., Gallor L. (1992). Biology of the syndecans: A family of transmembrane heparan sulfate proteoglycans. Annu. Rev. Cell Biol..

[47] Fransson L.-Å. (2003). Glypicans. Int. J. Biochem. Cell Biol..

[48] Lembo D., Donalisio M., Laine C., Cagno V., Civra A., Bianchini E.P., Zeghbib N., Bouchemal K. (2014). Auto-associative heparin nanoassemblies: A biomimetic platform against the heparan sulfate-dependent viruses HSV-1, HSV-2, HPV-16 and RSV. Eur. J. Pharm. Biopharm..

[49] Schäfer G., Blumenthal, Melissa J., Katz A.A. (2015). Interaction of human tumor viruses with host cell surface receptors and cell entry. Viruses.

[50] Tyagi M., Rusnati M., Presta M., Giacca M. (2001). Internalization of HIV-1 Tat Requires Cell Surface Heparan Sulfate Proteoglycans. J. Biol. Chem..

[51] Kalia M., Chandra V., Rahman S.A., Sehgal D., Jameel S. (2009). Heparan Sulfate Proteoglycans Are Required for Cellular Binding of the Hepatitis E Virus ORF2 Capsid Protein and for Viral Infection. J. Virol..

[52] Xu Y., Martinez P., Séron K., Luo G., Allain F., Dubuisson J., Belouzard S. (2015). Characterization of Hepatitis C Virus Interaction with Heparan Sulfate Proteoglycans. J. Virol..

[53] Summerford C., Samulski R.J. (1998). Membrane-Associated Heparan Sulfate Proteoglycan Is a Receptor for Adeno-Associated Virus Type 2 Virions. J. Virol..

[54] Hilgard P., Stockert R. (2000). Heparan sulfate proteoglycans initiate dengue virus infection of hepatocytes. Hepatology.

[55] Schulze A., Gripon P., Urban S. (2007). Hepatitis B virus infection initiates with a large surface protein-dependent binding to heparan sulfate proteoglycans. Hepatology.

[56] Shieh M.T., WuDunn D., Montgomery R.I., Esko J.D., Spear P.G. (1992). Cell surface receptors for herpes simplex virus are heparan sulfate proteoglycans. J. Cell Biol..

[57] Culp T.D., Budgeon L.R., Christensen N.D. (2006). Human papillomaviruses bind a basal extracellular matrix component secreted by keratinocytes which is distinct from a membrane-associated receptor. Virology.

[58] Culp T.D., Budgeon L.R., Marinkovich M.P., Meneguzzi G., Christensen N.D. (2006). Keratinocyte-Secreted Laminin 5 Can Function as a Transient Receptor for Human Papillomaviruses by Binding Virions and Transferring Them to Adjacent Cells. J. Virol..

[59] Cerqueira C., Ventayol P.S., Vogeley C., Schelhaas M. (2015). Kallikrein-8 Proteolytically Processes Human Papillomaviruses in the Extracellular Space To Facilitate Entry into Host Cells. J. Virol..

[60] Bienkowska-Haba M., Patel H.D., Sapp M. (2009). Target Cell Cyclophilins Facilitate Human Papillomavirus Type 16 Infection. Pathogens.

[61] Richards R.M., Lowy D.R., Schiller J.T., Day P.M. (2006). Cleavage of the papillomavirus minor capsid protein, L2, at a furin consensus site is necessary for infection. Proc. Natl. Acad. Sci. USA.

[62] Selinka H.-C., Florin L., Patel H.D., Freitag K., Schmidtke M., Makarov V.A., Sapp M. (2007). Inhibition of Transfer to Secondary Receptors by Heparan Sulfate-Binding Drug or Antibody Induces Noninfectious Uptake of Human Papillomavirus. J. Virol..

[63] Day P.M., Lowy D.R., Schiller J.T. (2008). Heparan Sulfate-Independent Cell Binding and Infection with Furin-Precleaved Papillomavirus Capsids. J. Virol..

[64] Schelhaas M., Shah B., Holzer M., Blattmann P., Kühling L., Day P.M., Schiller J.T., Helenius A. (2012). Entry of human papillomavirus type 16 by actin-dependent, clathrin- and lipid raft-independent endocytosis. PLoS Pathog..

[65] Spoden G., Freitag K., Husmann M., Boller K., Sapp M., Lambert C., Florin L. (2008). Clathrin- and Caveolin-Independent Entry of Human Papillomavirus type 16-Involvement of Tetraspanin-Enriched Microdomains (TEMs). PLoS ONE.

[66] Bannach C., Brinkert P., Kühling L., Greune L., Schmidt M.A., Schelhaas M. (2020). Epidermal Growth Factor Receptor and Abl2 Kinase Regulate Distinct Steps of Human Papillomavirus 16 Endocytosis. J. Virol..

[67] Fast L.A., Mikuličić S., Fritzen A., Schwickert J., Boukhallouk F., Hochdorfer D., Sinzger C., Suarez H., Monk P.N., Yáñez-Mó M. (2018). Inhibition of tetraspanin functions impairs human papillomavirus and cytomegalovirus infections. Int. J. Mol. Sci..

[68] Hampe L., Boukhallouk F., Schneider M.A., Spoden G.A., Negwer I., Koynov K., Kast W.M., Florin L. (2016). The Cytoskeletal Adaptor Obscurin-Like 1 Interacts with the Human Papillomavirus 16 ( HPV16 ) Capsid Protein L2 and Is Required for. J. Virol..

[69] Dziduszko A., Ozbun M.A. (2013). Annexin A2 and S100A10 Regulate Human Papillomavirus Type 16 Entry and Intracellular Trafficking in Human Keratinocytes. J. Virol..

[70] Spoden G., Kühling L., Cordes N., Frenzel B., Sapp M., Boller K., Florin L. (2013). Human Papillomavirus Types 16, 18, and 31 Share Similar Endocytic Requirements for Entry. J. Virol..

[71] Bergant M., Ozbun M.A., Campos S.K., Myers M.P., Banks L. (2012). Human Papillomavirus L2 Facilitates Viral Escape from Late Endosomes via Sorting Nexin 17. Traffic.

[72] Smith J.L., Campos S.K., Wandinger-Ness A., Ozbun M.A. (2008). Caveolin-1-Dependent Infectious Entry of Human Papillomavirus Type 31 in Human Keratinocytes Proceeds to the Endosomal Pathway for pH-Dependent Uncoating. J. Virol..

[73] Gräßel L., Fast L.A., Scheffer K.D., Boukhallouk F., Overduin M., Berditchevski F., Florin L. (2016). The CD63-Syntenin-1 Complex Controls Post-Endocytic Trafficking of Oncogenic Human Papillomaviruses. Sci. Rep..

[74] Taylor J.R., Fernandez D.J., Thornton S.M., Skeate J.G., Lühen K.P., Da Silva D.M., Langen R., Kast W.M. (2018). Heterotetrameric annexin A2/S100A10 (A2t) is essential for oncogenic human papillomavirus trafficking and capsid disassembly, and protects virions from lysosomal degradation. Sci. Rep..

[75] Selinka H.C., Giroglou T., Sapp M. (2002). Analysis of the infectious entry pathway of human papillomavirus type 33 pseudovirions. Virology.

[76] DiGiuseppe S., Bienkowska-Haba M., Guion L.G.M., Keiffer T.R., Sapp M. (2017). Human Papillomavirus Major Capsid Protein L1 Remains Associated with the Incoming Viral Genome throughout the Entry Process. J. Virol..

[77] Yan H., Foo S.S., Chen W., Yoo J.S., Shin W.J., Wu C., Jung J.U. (2019). Efficient inhibition of human papillomavirus infection by L2 minor capsid-derived lipopeptide. Am. Soc. Microbiol..

[78] Zhang P., Monteiro da Silva G., Deatherage C., Burd C., DiMaio D. (2018). Cell-Penetrating Peptide Mediates Intracellular Membrane Passage of Human Papillomavirus L2 Protein to Trigger Retrograde Trafficking. Cell.

[79] Bronnimann M.P., Chapman J.A., Park C.K., Campos S.K. (2013). A Transmembrane Domain and GxxxG Motifs within L2 Are Essential for Papillomavirus Infection. J. Virol..

[80] Inoue T., Zhang P., Zhang W., Bingham K.G., Dupzyk A., Dimaio D., Tsai B. (2018). γ-Secretase promotes membrane insertion of the human papillomavirus L2 capsid protein during virus infection. J. Cell Biol..

[81] Popa A., Zhang W., Harrison M.S., Goodner K., Kazakov T., Goodwin E.C., Lipovsky A., Burd C.G., DiMaio D. (2015). Direct Binding of Retromer to Human Papillomavirus Type 16 Minor Capsid Protein L2 Mediates Endosome Exit during Viral Infection. PLoS Pathog..

[82] Siddiqa A., Massimi P., Pim D., Broniarczyk J., Banks L. (2018). Human Papillomavirus 16 Infection Induces VAP-Dependent Endosomal Tubulation. J. Virol..

[83] Pyeon D., Pearce S.M., Lank S.M., Ahlquist P., Lambert P.F. (2009). Establishment of Human Papillomavirus Infection Requires Cell Cycle Progression. PLoS Pathog..

[84] Aydin I., Weber S., Snijder B., Samperio Ventayol P., Kühbacher A., Becker M., Day P.M., Schiller J.T., Kann M., Pelkmans L. (2014). Large Scale RNAi Reveals the Requirement of Nuclear Envelope Breakdown for Nuclear Import of Human Papillomaviruses. PLoS Pathog..

[85] Calton C.M., Bronnimann M.P., Manson A.R., Li S., Chapman J.A., Suarez-Berumen M., Williamson T.R., Molugu S.K., Bernal R.A., Campos S.K. (2017). Translocation of the papillomavirus L2/vDNA complex across the limiting membrane requires the onset of mitosis. PLoS Pathog..

[86] Broniarczyk J., Massimi P., Bergant M., Banks L. (2015). Human Papillomavirus Infectious Entry and Trafficking Is a Rapid process. J. Virol..

[87] Schneider M.A., Spoden G.A., Florin L., Lambert C. (2011). Identification of the dynein light chains required for human papillomavirus infection. Cell. Microbiol..

[88] Aydin I., Villalonga-Planells R., Greune L., Bronnimann M.P., Calton C.M., Becker M., Lai K., Campos S.K., Schmidt A., Schelhaas M. (2017). A central region in the minor capsid protein of papillomaviruses facilitates viral genome tethering and membrane penetration for mitotic nuclear entry. PLoS Pathog..

[89] Day P.M., Baker C.C., Lowy D.R., Schiller J.T. (2004). Establishment of papillomavirus infection is enhanced by promyelocytic leukemia protein (PML) expression. Proc. Natl. Acad. Sci. USA.

[90] Rose R.C., Bonnez W., Reichman R.C., Garcea R.L. (1993). Expression of Human Papillomavirus Type 11 Protein in Insect Cells: In Vivo and In Vitro Assembly of Viruslike Particles. J. Virol..

[91] Zhou J., Sun X., Louis K., Frazer I.H. (1994). Interaction of Human Papillomavirus (HPV) Type 16 Capsid Proteins with HPV DNA Requires an Intact L2 N-Terminal Sequence. J. Virol..

[92] Buck C.B., Pastrana D.V., Lowy D.R., Schiller J.T. (2005). Generation of HPV pseudovirions using transfection and their use in neutralization assays. Methods Mol. Med..

[93] Schäfer G., Kabanda S., Van Rooyen B., Bergant M., Banks L., Parker M.I. (2013). The role of inflammation in HPV infection of the Oesophagus. BMC Cancer.

[94] Drobni P., Mistry N., McMillan N., Evander M. (2003). Carboxy-fluorescein diacetate, succinimidyl ester labeled papillomavirus virus-like particles fluoresce after internalization and interact with heparan sulfate for binding and entry. Virology.

[95] Joyce J.G., Tung J.S., Przysiecki C.T., Cook J.C., Lehman E.D., Sands J.A., Jansen K.U., Keller P.M. (1999). The L1 major capsid protein of human papillomavirus type 11 recombinant virus-like particles interacts with heparin and cell-surface glycosaminoglycans on human keratinocytes. J. Biol. Chem..

[96] Day P.M., Thompson C.D., Buck C.B., Pang Y.S., Lowy D.R., Schiller J.T. (2007). Neutralization of Human Papillomavirus with Monoclonal Antibodies Reveals Different Mechanisms of Inhibition. J. Virol..

[97] Cruz L., Meyers C. (2013). Differential Dependence on Host Cell Glycosaminoglycans for Infection of Epithelial Cells by High-Risk HPV Types. PLoS ONE.

[98] Patterson N.A., Smith J.L., Ozbun M.A. (2005). Human Papillomavirus Type 31b Infection of Human Keratinocytes Does Not Require Heparan Sulfate. J. Virol..

[99] Cerqueira C., Liu Y., Kühling L., Chai W., Hafezi W., Van Kuppevelt T.H., Kühn J.E., Feizi T., Schelhaas M. (2013). Heparin increases the infectivity of Human Papillomavirus Type 16 independent of cell surface proteoglycans and induces L1 epitope exposure. Cell. Microbiol..

[100] Piret J., Lamontagne J., Bestman-smith J., Roy S., Omar R.F., Juha J., Gourde P., Bergeron M.G. (2000). In Vitro and In Vivo Evaluations of Sodium Lauryl Sulfate and Dextran Sulfate as Microbicides against Herpes Simplex and Human Immunodeficiency Viruses. J. Clin. Microbiol..

[101] Herold B.C., Siston A., Bremer J., Kirkpatrick R., Wilbanks G., Fugedi P., Peto C., Cooper M. (1997). Sulfated Carbohydrate Compounds Prevent Microbial Adherence by Sexually Transmitted Disease Pathogens. Antimicrob. Agents Chemother..

[102] Herold B.C., Bourne N., Marcellino D., Kirkpatrick R., Strauss D.M., Zaneveld L.J.D., Waller D.P., Anderson R.A., Chany C.J., Barham B.J. (2000). Poly (Sodium 4-Styrene Sulfonate): An Effective Candidate Topical Antimicrobial for the Prevention of Sexually Transmitted Diseases. J. Infect. Dis..

[103] Lembo D., Donalisio M., Rusnati M., Bugatti A., Cornaglia M., Cappello P., Giovarelli M., Oreste P., Landolfo S. (2008). Sulfated K5 Escherichia coli Polysaccharide Derivatives as Wide-Range Inhibitors of Genital Types of Human Papillomavirus. Antimicrob. Agents Chemother..

[104] Wang S., Lu Z., Wang S., Liu W., Gao J., Tian L., Wang L., Zhang X., Zhao X., Wang W. (2020). The inhibitory effects and mechanisms of polymannuroguluronate sulfate against human papillomavirus infection in vitro and in vivo. Carbohydr. Polym..

[105] Sinha S., Astani A., Ghosh T., Schnitzler P., Ray B. (2010). Phytochemistry Polysaccharides from Sargassum tenerrimum: Structural features, chemical modification and anti-viral activity. Phytochemistry.

[106] Wu L., Wang W., Zhang X., Zhao X., Yu G. (2016). Anti-HBV activity and mechanism of marine-derived polyguluronate sulfate (PGS) in vitro. Carbohydr. Polym..

[107] Thi T., Thuy T., Minh B., Thi T., Van T., Van Quang N., Cam H., Zheng Y., Seguin-devaux C., Mi B. (2015). Anti-HIV activity of fucoidans from three brown seaweed species. Carbohydr. Polym..

[108] Buck C.B., Thompson C.D., Lowy D.R., Roberts J.N., Mu M., Schiller J.T. (2006). Carrageenan Is a Potent Inhibitor of Papillomavirus Infection. PLoS Pathog..

[109] Calagna G., Maranto M., Paola C., Capra G., Perino A., Chiantera V., Cucinella G., Calagna G., Maranto M., Paola C. (2020). ‘Secondary prevention’ against female HPV infection: Literature review of the role of carrageenan. Expert Rev. Anti. Infect. Ther..

[110] Rodríguez A., Kleinbeck K., Mizenina O., Kizima L., Levendosky K., Jean-Pierre N., Villegas G., Ford B.E., Cooney M.L., Teleshova N. (2014). In vitro and in vivo evaluation of two carrageenan-based formulations to prevent HPV acquisition. Antivir. Res..

[111] Novetsky A.P., Keller M.J., Gradissimo A., Chen Z., Morgan S.L., Xue X., Strickler H.D., Fernández-romero J.A., Burk R., Einstein M.H. (2016). Gynecologic Oncology In vitro inhibition of human papillomavirus following use of a carrageenan-containing vaginal gel. Gynecol. Oncol..

[112] Marais D., Gawarecki D., Allan B., Ahmed K., Altini L., Cassim N., Hoffman M., Ramjee G., Williamson A. (2011). The effectiveness of Carraguard, a vaginal microbicide, in protecting women against high-risk human papillomavirus infection. Antivir. Ther..

[113] Skoler-Karpoff S., Ramjee G., Ahmed K., Altini L., Plagianos M.G., Friedland B., Govender S., Town C., Town C. (2008). Efficacy of Carraguard for prevention of HIV infection in women in South Africa: A randomised, double-blind, placebo-controlled trial. Lancet.

[114] Magnan S., Tota J.E., Burchell A.N., Schiller J.T., Ferenczy A., Franco E.L., Study C. (2019). Efficacy of a Carrageenan gel Against Transmission of Cervical HPV (CATCH): Interim analysis of a randomized, double-blind, placebo-controlled, phase 2B trial. Clin. Microbiol. Infect..

[115] Perino A., Consiglio P., Maranto M., Franciscis P.D.E., Marci R. (2019). Impact of a new carrageenan-based vaginal microbicide in a female population with genital HPV-infection: First experimental results. Eur. Rev. Med. Pharmacol. Sci..

[116] Soria-Martinez L., Bauer S., Giesler M., Schelhaas S., Materlik J., Janus K., Pierzyna P., Becker M., Snyder N.L., Hartmann L. (2020). Prophylactic Antiviral Activity of Sulfated Glycomimetic Oligomers and Polymers. JACS.

[117] Zhang G., Ye X. (2018). Synthetic Glycans and Glycomimetics: A Promising Alternative to Natural Polysaccharides. Chemistry (Easton).

[118] Schmidtke M., Karger A., Meerbach A., Egerer R., Stelzner A., Makarov V. (2003). Binding of a N,N’-bisheteryl derivative of dispirotripiperazine to heparan sulfate residues on the cell surface specifically prevents infection of viruses from different families. Virology.

[119] Spoden G.A., Besold K., Krauter S., Plachter B., Hanik N., Kilbinger A.F.M., Lambert C., Florin L. (2012). Polyethylenimine Is a Strong Inhibitor of Human Papillomavirus and Cytomegalovirus Infection. Antimicrob. Agents Chemother..

[120] Schermant D., Demeneixt B., Behr J. (1995). A versatile vector for gene and oligonucleotide transfer into cells in culture and in vivo: Polyethylenimine. Proc. Natl. Acad. Sci. USA.

[121] Bellamy W., Wakabayashi H., Takase M., Kawase K., Shimamura S., Tomita M. (1993). Killing of Candida albicans by lactoferricin B, a potent antimicrobial peptide derived from the N-terminal region of bovine lactoferrin. Med. Microbiol. Imunnol..

[122] Andersen J.H., Jenssen H., Gutteberg T.J. (2003). Lactoferrin and lactoferricin inhibit Herpes simplex 1 and 2 infection and exhibit synergy when combined with acyclovir. Antivir. Res..

[123] Sandvik K., Gutteberg T.J., Andersen J.H. (2004). Anti-HSV Activity of Lactoferrin and Lactoferricin is Dependent on the Presence of Heparan Sulphate at the Cell Surface. J. Med. Virol..

[124] Harmsen M.C., Swart P.J., De Béthune M., Pauwels R., The S., Diseases I., Aug N., Harmsen M.C., Swart P.J., Pauwels R. (1995). Antiviral Effects of Plasma and Milk Proteins: Lactoferrin Shows Potent Activity against Both Human Immunodeficiency Virus and Human Cytomegalovirus Replication in vitro. J. Infect. Dis..

[125] Ikeda M., Sugiyama K., Tanaka T., Tanaka K., Sekihara H., Shimotohno K., Kato N. (1998). Lactoferrin Markedly Inhibits Hepatitis C Virus Infection in Cultured Human Hepatocytes. Biochemical.

[126] Longhi G., Pietropaolo V., Mischitelli M., Longhi C., Pia M., Marchetti M., Tinari A., Valenti P., Marta A., Seganti L. (2006). Lactoferrin inhibits early steps of human BK polyomavirus infection. Antivir. Res..

[127] Drobni P., Näslund J., Evander M. (2004). Lactoferrin inhibits human papillomavirus binding and uptake in vitro. Antivir. Res..

[128] Mistry N., Drobni P., Näslund J., Sunkari V.G., Jenssen H., Evander M. (2007). The anti-papillomavirus activity of human and bovine lactoferricin. Antivir. Res..

[129] Gifford J.L., Hunter H.N., Vogel H.J. (2005). Lactoferricin: A lactoferrin-derived peptide with antimicrobial, antiviral, antitumor and immunological properties. Cell. Mol. Life Sci..

[130] Wu H., Monroe D.M., Church F.C. (1995). Characterization of the Glycosaminoglycan-Binding Region of Lactoferrin. Arch. Biochem. Biophys..

[131] Marchetti M., Superti F., Grazia M., Paola A., Rossi P. (1999). Inhibition of poliovirus type 1 infection by iron-, manganese- and zinc-saturated lactoferrin. Med. Microbiol. Imunnol..

[132] Bourne N., Stanberry L.R., Kern E.R., Holan G., Matthews B. (2000). Dendrimers, a New Class of Candidate Topical Microbicides with Activity against Herpes Simplex Virus Infection. Antimicrob. Agents Chemother..

[133] Donalisio M., Rusnati M., Civra A., Bugatti A., Allemand D., Pirri G., Giuliani A., Landolfo S., Lembo D. (2010). Identification of a dendrimeric heparan sulfate-binding peptide that inhibits infectivity of genital types of human papillomaviruses. Antimicrob. Agents Chemother..

[134] Virtanen I., Lehto V., Lehtonen E., Vartio T., Stenman S., Kurki P., Wager O., Small J.V., Dahl D., Badley R.A. (1981). Expression of intermediate filaments in cultured cells. J. Cell Sci..

[135] Brown M.J., Hallam J.A., Colucci-Guyon E., Shaw S. (2001). Rigidity of circulating lymphocytes is primarily conferred by vimentin intermediate filaments. J. Immunol..

[136] Azumi N., Battifora H. (1987). The distribution of vimentin and keratin in epithelial and nonepithelial neoplasms. A comprehensive immunohistochemical study on formalin- and alcohol-fixed tumors. Am. J. Clin. Pathol..

[137] Herrmann H., Bär H., Kreplak L., Strelkov S.V., Aebi U. (2007). Intermediate filaments: From cell architecture to nanomechanics. Nat. Rev. Mol. Cell Biol..

[138] Ivaska J., Pallari H.M., Nevo J., Eriksson J.E. (2007). Novel functions of vimentin in cell adhesion, migration, and signaling. Exp. Cell Res..

[139] Moisan E., Girard D. (2006). Cell surface expression of intermediate lament proteins vimentin and lamin B1 in human neutrophil spontaneous apoptosis. J. Leukoc. Biol..

[140] Bhattacharya R., Gonzalez A.M., DeBiase P.J., Trejo H.E., Goldman R.D., Flitney F.W., Jones J.C.R. (2009). Recruitment of vimentin to the cell surface by β3 integrin and plectin mediates adhesion strength. J. Cell Sci..

[141] Mor-Vaknin N., Punturieri A., Sitwala K., Markovitz D.M. (2003). Vimentin is secreted by activated macrophages. Nat. Cell Biol..

[142] Shigyo M., Tohda C. (2016). Extracellular vimentin is a novel axonal growth facilitator for functional recovery in spinal cord-injured mice. Sci. Rep..

[143] Das S., Ravi V., Desai A. (2011). Japanese encephalitis virus interacts with vimentin to facilitate its entry into porcine kidney cell line. Virus Res..

[144] Liang J., Yu C., Liao C., Lin Y. (2011). Vimentin binding is critical for infection by the virulent strain of Japanese encephalitis virus. Cell. Microbiol..

[145] Miller M.S., Hertel L. (2009). Onset of Human Cytomegalovirus Replication in Fibroblasts Requires the Presence of an Intact Vimentin Cytoskeleton. J. Virol..

[146] Du N., Cong H., Tian H., Zhang H., Zhang W., Song L., Tien P. (2014). Cell Surface Vimentin Is an Attachment Receptor for Enterovirus 71. J. Virol..

[147] Kokuba H., Aurelian L., Neurath A.R. (1998). 3-Hydroxyphthaloyl β-lactoglobulin. IV. Antiviral activity in the mouse model of genital herpesvirus infection. Antivir. Chem. Chemother..

[148] Neurath A.R., Strick N., Li Y.Y. (1998). 3-Hydroxyphthaloyl β-lactoglobulin. III. Antiviral activity against herpesviruses. Antivir. Chem. Chemother..

[149] Li L., He L., Tan S., Guo X., Lu H., Qi Z., Pan C., An X., Jiang S., Liu S. (2010). 3-hydroxyphthalic anhydride-modified chicken ovalbumin exhibits potent and broad anti-HIV-1 activity: A potential microbicide for preventing sexual transmission of HIV-1. Antimicrob. Agents Chemother..

[150] Lu L., Yang X., Li Y., Jiang S. (2013). Chemically modified bovine beta-lactoglobulin inhibits human papillomavirus infection. Microbes Infect..

[151] Hua C., Zhu Y., Wu C., Si L., Wang Q., Sui L., Jiang S. (2019). The underlying mechanism of 3-hydroxyphthalic anhydride-modified bovine beta-lactoglobulin to block human papillomavirus entry into the host cell. Front. Microbiol..

[152] Guo X., Qiu L., Wang Y., Wang Y., Wang Q., Song L., Li Y., Huang K., Du X., Fan W. (2016). A randomized open-label clinical trial of an anti-HPV biological dressing (JB01-BD) administered intravaginally to treat high-risk HPV infection. Microbes Infect..

[153] Woodham A.W., da Silva D.M., Skeate J.G., Raff A.B., Ambroso M.R., Brand H.E., Isas J.M., Langen R., Kast W.M. (2012). The S100A10 subunit of the annexin A2 heterotetramer facilitates L2-mediated human papillomavirus infection. PLoS ONE.

[154] Woodham A.W., Taylor J.R., Jimenez A.I., Skeate J.G., Schmidt T., Brand H.E., Da Silva D.M., Martin Kast W. (2014). Small molecule inhibitors of the annexin A2 heterotetramer prevent human papillomavirus type 16 infection. J. Antimicrob. Chemother..

[155] Latysheva N., Muratov G., Rajesh S., Padgett M., Hotchin N.A., Overduin M., Berditchevski F. (2006). Syntenin-1 Is a New Component of Tetraspanin-Enriched Microdomains: Mechanisms and Consequences of the Interaction of Syntenin-1 with CD63. Mol. Cell. Biol..

[156] Scheffer K.D., Berditchevski F., Florin L. (2014). The Tetraspanin CD151 in Papillomavirus Infection. Viruses.

[157] Lipovsky A., Erden A., Kanaya E., Zhang W., Crite M., Bradfield C., Macmicking J., Dimaio D., Schoggins J.W., Iwasaki A. (2017). The cellular endosomal protein stannin inhibits intracellular trafficking of human papillomavirus during virus entry. J. Gen. Virol..

[158] Müller K.H., Spoden G.A., Scheffer K.D., Brunnhöfer R., De Brabander J.K., Maier M.E., Florin L., Muller C.P. (2014). Inhibition by cellular vacuolar atpase impairs human papillomavirus uncoating and infection. Antimicrob. Agents Chemother..

[159] Forgac M. (1999). Structure and Properties of the Vacuolar (H+)-ATPases. J. Biol. Chem..

[160] Lebreton S., Jaunbergs J., Roth M.G., Ferguson D.A., De Brabander J.K. (2008). Evaluating the potential of Vacuolar ATPase inhibitors as anticancer agents and multigram synthesis of the potent salicylihalamide analog saliphenylhalamide. Bioorg. Med. Chem. Lett..

[161] Buck C.B., Day P.M., Thompson C.D., Lubkowski J., Lu W., Lowy D.R., Schiller J.T. (2006). Human α-defensins block papillomavirus infection. Proc. Natl. Acad. Sci. USA.

[162] Wiens M.E., Smith J.G. (2015). Papillomavirus 16 L2 To Block Infection. Virology.

[163] Karanam B., Peng S., Li T., Buck C., Day P.M., Roden R.B.S. (2010). Papillomavirus Infection Requires γ Secretase. J. Virol..

[164] Zhang W., Kazakov T., Popa A., DiMaio D. (2014). Vesicular Trafficking of Incoming Human Papillomavirus 16 to the Golgi Apparatus and Endoplasmic Reticulum Requires γ-Secretase Activity. MBio.

[165] Huang H.S., Buck C.B., Lambert P.F. (2010). Inhibition of gamma secretase blocks HPV infection. Virology.

[166] Kwak K., Jiang R., Wang J.W., Jagu S., Kirnbauer R., Roden R.B.S. (2014). Impact of Inhibitors and L2 Antibodies upon the Infectivity of Diverse Alpha and Beta Human Papillomavirus Types. PLoS ONE.

[167] Zhang P., Moreno R., Lambert P.F., DiMaio D. (2020). Cell-penetrating peptide inhibits retromer-mediated human papillomavirus trafficking during virus entry. Proc. Natl. Acad. Sci. USA.

[168] Ito M., Baba M., Sato A., Pauwels R., De Clercq E., Shigeta S. (1987). Inhibitory effect of dextran sulfate and heparin on the replication of human immunodeficiency virus (HIV) in vitro. Antivir. Res..

[169] Rider C.C. (1997). The potential for heparin and its derivatives in the therapy and prevention of HIV-1 infection. Glycoconj. J..

[170] Thompson III G., Lawrence V.A., Crawford G.E. (2007). HIV/AIDS HIV Infection Increases the Risk of Heparin-Induced Thrombocytopenia. Clin. Infect. Dis..

[171] Groveman M.D.S. (1996). Inhibition of HIV-1 infectivity by low molecular weight heparin: Results of in vitro studies and a pilot clinical trial in patients with advanced AIDS. Int. J. Clin. Lab. Res..

[172] Compton T., Nowlin D.M., Cooper N.R. (1993). Initiation of Human Cytomegalovirus Infection Requires Initial Interaction with Cell Surface Heparan Sulfate. Virology.

[173] Anderson R.A., Feathergill K., Diao X., Cooper M., Kirkpatrick R., Spear P., Waller D.P., Chany C., Doncel G.F., Herold B. (2000). Evaluation of Poly(Styrene-4-Sulfonate) as a Preventive Agent for Conception and Sexually Transmitted Diseases. J. Androl..

[174] Yamada T., Ogamo A., Saito T., Watanabe J., Uchiyama H. (1997). Preparation and anti-HIV activity of low- molecular-weight carrageenans and their sulfated derivatives. Carbohydr. Polym..

[175] Phillips D.M. (1997). Vaginal Formulations of Carrageenan Protect Mice from Herpes Simplex Virus Infection. Clin. Diagn. Lab. Immunol..

[176] Anderson J., Osbakk S., Vorland L., T T., TJ G. (2001). Lactoferrin and cyclic lactoferricin inhibit the entry of human cytomegalovirus into human fibroblasts. Antivir. Res..

[177] Van der Strate B., Beljaars L., Molema G., Harmsen M., Meijer D. (2001). Antiviral activities of lactoferrin. Antivir. Res..

[178] Hara K., Ikeda M., Saito S., Matsumoto S., Numata K., Kato N., Tanaka K., Sekihara H. (2002). Lactoferrin inhibits hepatitis B virus infection in cultured human hepatocytes. Hepatol. Res..

[179] Bon I., Lembo D., Rusnati M., Clò A., Morini S., Miserocchi A., Bugatti A., Girogolon S., Musumeci G., Landolfo S. (2013). Peptide-Derivatized SB105-A10 Dendrimer Inhibits the Infectivity of R5 and X4 HIV-1 Strains in Primary PBMCs and Cervicovaginal Histocultures. PLoS ONE.

[180] Luganini A., Giuliani A., Pirri G., Pizzuto L., Landolfo S., Gribaudo G. (2010). Peptide-derivatized dendrimers inhibit human cytomegalovirus infection by blocking virus binding to cell surface heparan sulfate. Antivir. Res..

[181] Sep D., Ce R., Jim L. (2017). Mechanistic Studies of Viral Entry: An Overview of Dendrimer-Based Microbicides As Entry Inhibitors Against Both HIV and HSV-2 Overlapped Infections. Med. Recearch Rev..

[182] Luganini A., Nicoletto S.F., Pizzuto L., Pirri G., Giuliani A., Landolfo S., Gribaudo G. (2011). Inhibition of Herpes Simplex Virus Type 1 and Type 2 Infections by Peptide-Derivatized Dendrimers. Antimicrob. Agents Chemother..

[183] Neurath A.R., Debnath A.K., Strick N., Li Y.-Y., Lin K., Jiang S. (1995). Blocking of CD4 cell receptors for the human immunodeficiency virus type 1 (HIV-1) by chemically modified bovine milk proteins: Potential for AIDS prophylaxis. J. Mol. Recognit..

[184] Hazrati E., Galen B., Lu W., Wang W., Ouyang Y., Keller M.J., Lehrer R.I., Herold B.C. (2006). Human α- and β-Defensins Block Multiple Steps in Herpes Simplex Virus Infection. J. Immunol..

[185] Wiens M.E., Smith J.G. (2017). alpha-Defensin HD5 Inhibits Human Papillomavirus 16 Infection via Capsid Stabilization and Redirection to the Lysosome. Am. Soc. Microbiol..

